# Navigating the Immunological Crossroads: Mesenchymal Stem/Stromal Cells as Architects of Inflammatory Harmony in Tissue-Engineered Constructs

**DOI:** 10.3390/bioengineering11050494

**Published:** 2024-05-16

**Authors:** Saeed Farzamfar, Luciana Melo Garcia, Mahya Rahmani, Stephane Bolduc

**Affiliations:** 1 Centre de Recherche en Organogénèse Expérimentale/LOEX, Regenerative Medicine Division, CHU de Québec-Université Laval Research Center, Québec, QC G1V 4G2, Canada; saeed.farzamfar@crchudequebec.ulaval.ca (S.F.); mahya.rahmani@gmail.com (M.R.); 2 Department of Medicine, Université Laval, Québec, QC G1V 0A6, Canada; luciana.melo-garcia@crchudequebec.ulaval.ca; 3 Hematology-Oncology Service, CHU de Québec—Université Laval, Québec, QC G1V 0A6, Canada; 4 Department of Surgery, Faculty of Medicine, Université Laval, Québec, QC G1V 0A6, Canada

**Keywords:** immune system, tissue engineering, mesenchymal stem/stromal cells, tissue regeneration

## Abstract

In the dynamic landscape of tissue engineering, the integration of tissue-engineered constructs (TECs) faces a dual challenge—initiating beneficial inflammation for regeneration while avoiding the perils of prolonged immune activation. As TECs encounter the immediate reaction of the immune system upon implantation, the unique immunomodulatory properties of mesenchymal stem/stromal cells (MSCs) emerge as key navigators. Harnessing the paracrine effects of MSCs, researchers aim to craft a localized microenvironment that not only enhances TEC integration but also holds therapeutic promise for inflammatory-driven pathologies. This review unravels the latest advancements, applications, obstacles, and future prospects surrounding the strategic alliance between MSCs and TECs, shedding light on the immunological symphony that guides the course of regenerative medicine.

## 1. Introduction

Tissue engineering, an innovative area within regenerative medicine, seeks to repair or substitute damaged tissues through the development of scaffolds that imitate the structure and function of natural tissues. Yet, the effective assimilation of these tissue-engineered constructs (TECs) frequently faces hurdles presented by inflammatory reactions. Inflammation, a multifaceted biological mechanism, is essential for tissue regeneration but can also be harmful if not carefully controlled [1,2]. Upon implantation, scaffolds trigger an immediate innate immune response. This response involves the release of pro-inflammatory cytokines, chemokines, and the recruitment of immune cells [3,4]. While this acute inflammation is essential for initiating tissue repair, prolonged or excessive inflammation can lead to fibrosis, rejection, and impaired tissue regeneration [2,5,6]. Mesenchymal stem/stromal cells (MSCs) possess unique immunomodulatory properties that can be harnessed to regulate this inflammatory response [7]. These cells have been widely studied due to their remarkable ability to modulate the function of various immune cells, including macrophages [8], T cells [9], and B cells [10]. Through the secretion of anti-inflammatory factors such as interleukin-10 (IL-10) and transforming growth factor-beta (TGF-β) [11,12], MSCs create an immunosuppressive microenvironment. Furthermore, MSCs can shift macrophages from a pro-inflammatory (M1) to an anti-inflammatory (M2) phenotype, promoting a microenvironment conducive to tissue repair and regeneration [13,14]. Incorporating MSCs into TECs presents a strategic approach to modulating inflammatory responses. The paracrine effects of these cells can be utilized to create a local microenvironment favoring tissue regeneration. Extracellular vesicles released from these cells may modulate the inflammatory responses at the implantation site [15,16]. This strategic integration of MSCs not only enhances the construct’s integration but also holds promise for treating various inflammatory-driven pathologies [17]. Ongoing research is required to refine techniques for optimal MSC delivery, dosage, and timing to precisely control the host immune response.

This review explores the latest advancements, applications, obstacles, and future outlooks pertaining to the immunomodulation of TECs through the utilization of MSC.

## 2. The Principles of Immunological Reactions to Scaffolds

The immune system is composed of cells that perform multiple physiological functions. It protects the body against tumor development and infections while also maintaining the homeostasis of various processes, including inflammation and healing. In the field of regenerative medicine, the immune system has historically been seen as detrimental, as immune cells can cause inflammation, fibrosis, and scaffold degradation [18]. However, more recently, researchers have been attempting to modify scaffold structures to regulate their interaction with the immune system and restore tissue function [19,20,21].

Tissue repair and regeneration is a complex stepwise process necessary to restore tissue function following damage [22]. Following an injury, an inflammatory response is triggered by Damage-Associated Molecular Patterns (DAMPs) released by injured cells, which leads to the subsequent recruitment, proliferation, and activation of both non-hematopoietic and hematopoietic cells. This healing process can result in either incomplete repair (known as scarring or fibrosis) or complete tissue restoration. The latter outcome depends on the extent and duration of the immune response and the involvement of various cells. Therefore, adequate involvement and precise regulation of the immune system are crucial for determining whether TECs will be rejected or integrated into the host tissue [23,24].

Fine-tuning the phases of immune response to TECs (protein adsorption, acute inflammation, chronic inflammation, or regeneration) by scaffold design can improve regeneration and reestablishment of tissue function following scaffold implantation [25]. 

**Figure 1:**
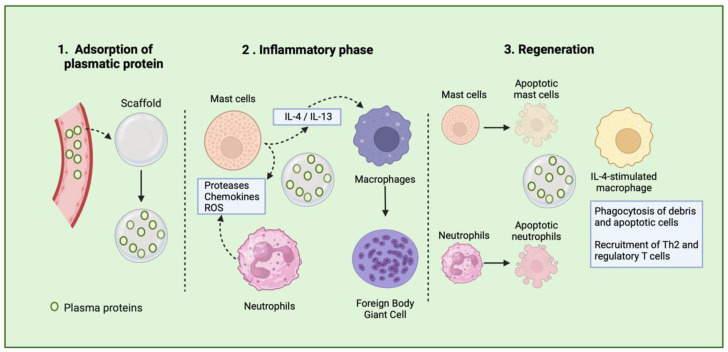
Schematic representation of tissue healing process after scaffold implantation.

Following implantation, plasma proteins (such as albumin, fibrinogen, complement, and fibronectin) bind to the scaffolds, a process called protein adsorption (Figure 1) [26,27]. The properties of biomaterials dictate how and which proteins bind to the scaffold’s surface. The type, quantity, and arrangement of the adsorbed proteins on the scaffold surface influence various immune responses, such as cell adhesion and activation. Cellular responses are initiated by these adsorbed proteins, which can activate receptors on the surface of immune cells, resulting in inflammatory responses, including macrophage adhesion. Additionally, non-cellular responses, such as complement activation, are triggered and contribute to leukocyte and platelet activation, promoting clot formation and inflammation [28,29]. Therefore, the composition of scaffold biomaterial determines the initial recruitment of immune cells, such as mast cells, neutrophils, and macrophages, which in turn determines the TEC’s rejection or tissue regeneration.

The second phase, or the inflammatory phase occurs following the recognition of the biomaterial as a foreign entity [25]. Initially, platelets and endothelial cells release histamine, cytokines, and leukotrienes at the site of the implanted TECs, resulting in the mobilization of neutrophils. Moreover, tissue-resident cells, such as macrophages and dendritic cells, detect the DAMPs released during scaffold implantation and attract neutrophils by producing the chemoattractant interleukin (IL-8) [22]. As the primary responders, neutrophils are recruited to the implant site within 72 h to defend against infections by generating cytotoxic products (such as proteases) and reactive oxygen species (ROS). They also release neutrophil extracellular traps (NETs) and produce IL-8, further attracting more neutrophils. This inflammatory response creates a degradative environment that can potentially degrade the surface of the biomaterial. Additionally, neutrophils produce NETs to eliminate pathogens; however, if not tightly regulated, this process may hinder the healing process. When present on the surface of biomaterials, NETs have been linked to promoting fibrosis [30,31]. However, neutrophils can also play a positive role in resolving fibrosis. Augmenting neutrophil quantities or boosting their production of matrix metalloproteinase (MMP) has been demonstrated to mitigate liver fibrosis [32]. The collective effect of neutrophils on fibrosis seems to hinge on the equilibrium between their inflammatory and anti-fibrotic capabilities. When neutrophil responses are dysregulated, it can shift the balance toward excessive fibrosis [33,34]. Therefore, new design strategies should be developed in order to drive neutrophils’ response to TECs.

Mast cells (MCs) are specialized immune cells found in tissues once they mature, playing crucial roles in the body’s reaction to biomaterials. A notable feature of MCs is their varied collection of granules, which are released upon activation. These granules play a pivotal role in subsequent inflammatory reactions, combating bacterial infections, neutralizing toxins, and responding to TECs [35].

Mast cells detect and react to biomaterial scaffolds via pattern recognition receptors and the high-affinity IgE receptor. Upon activation, they release various inflammatory substances like histamine, cytokines, and chemokines, influencing immune cell recruitment and polarization, notably macrophages. Research in mast cell-deficient mice indicates their absence disrupts the expected transition from M1 to M2 macrophages in response to decellularized biomaterial scaffolds. Male mice lacking mast cells exhibit prolonged pro-inflammatory M1 macrophage activity, while females show an early shift toward anti-inflammatory M2 macrophages. These sex-specific effects are reversible with mast cell adoptive transfer. Additionally, mast cells contribute to the foreign body response to implanted biomaterials, impacting scaffold integration or rejection [35,36].

Macrophages play a pivotal role in tissue inflammation, healing, and fibrosis. Tissue-resident macrophages have a complex origin involving multiple stages of hematopoietic development during embryogenesis. Initially, macrophages are formed during primitive hematopoiesis in the yolk sac alongside primitive erythroid and megakaryocyte progenitors, which also include microglia in the adult brain [37]. Following this, a second wave of development occurs, producing multipotent erythro-myeloid progenitors (EMPs) in the yolk sac, from which tissue-resident macrophages in various tissues (excluding the brain) emerge. Lastly, a third wave originates from hematopoietic stem cells (HSCs) originating in the aorta–gonad–mesonephros (AGM) region, migrating to the fetal liver, and giving rise to a durable population of macrophages persisting into adulthood [37,38,39].

In the initial stages of healing, macrophages bind to adsorbed proteins on the scaffold surface via integrin receptors. Subsequently, they attempt to engulf the biomaterial to which they are attached [40]. Interestingly, macrophages and neutrophils interact to influence the outcome of tissue remodeling following injury. The crosstalk between macrophages and neutrophils is particularly crucial for initiating tissue repair. Increased expression of phagocytic signals on the surface of dying neutrophils prompts the activation of macrophages. These macrophages then engulf the dying neutrophils, a process known as efferocytosis, which helps limit inflammation [41]. Furthermore, dying neutrophils secrete annexin-1, inhibiting interferon-gamma-associated responses that polarize macrophages into an inflammatory phenotype. Macrophages are also essential for neutrophilic reverse migration, a process characterized by the migration of neutrophils out of the tissues, causing dampening of inflammation [42,43]. Therefore, the acute phase of foreign body responses is characterized by the involvement of cells belonging to the innate immune system, often influenced by their interaction with scaffold biomaterials. The components of TECs, such as their supporting cells, incorporated bioactive agents, or the properties of the biomaterial, can guide these responses and ultimately lead to favorable responses to these constructs.

Macrophages adjust their functions based on the specific microenvironment in which they operate. They can exhibit a spectrum of activation phenotypes in response to various stimuli from their surroundings, including the properties of the biomaterial they adhere to and the nature of the tissue where the implant is located [44,45]. The chronic phase of the foreign body response is characterized by the formation of foreign body giant cells (FBGCs), which consist of fused macrophages located on the surface of the biomaterial implant. This process is primarily driven by cytokines such as IL-4 and IL-13, which are commonly found on biomaterial surfaces. The fusion of macrophages into FBGCs is also influenced by the composition of the scaffold and the proteins adsorbed onto its surface, both of which affect the phenotype of the FBGCs.

FBGCs play a crucial role in orchestrating subsequent immune responses by secreting cytokines that recruit and activate the adaptive immune system. Initially serving as inflammatory cells, FBGCs release degradation products like reactive oxygen species, degradative enzymes, and acid onto the biomaterial surface. Consequently, the biochemical properties of the biomaterial determine its susceptibility to biodegradation, which in turn affects the outcome of the implant. Upon stimulation with IL-4, FBGCs acquire a pro-remodeling phenotype that supports tissue repair by promoting a Th2 cell response [44,45,46]. This Th2 activation further enhances the IL-4-like cytokine release and reinforces the pro-remodeling phenotype in macrophages. These IL-4-like stimulated macrophages, associated with better scaffold outcomes, interact with other cells like fibroblasts and stem cells to promote tissue regeneration and functional implants [19]. Immune system action on biomaterials is a determinant factor in the outcomes of implants; thus, scaffold design aiming to modulate immune reactions is surging as a solution to improve tissue repair and regeneration.

TNF-α plays a crucial role in influencing the destiny and functional transformation of MSCs, either independently or in conjunction with various inflammatory agents. It can have contrasting impacts on MSCs, ranging from prompting MSC apoptosis to boosting their ability to combat tumors. Furthermore, the immunomodulatory and osteogenic differentiation capabilities of MSCs, as well as the composition of their exosomes or microvesicles, exhibit significant variation depending on factors such as the concentration of TNF-α used for stimulation, the timing of administration, and whether it is administered alone or in combination with other substances [47]. Treatment with IFN-γ prompts MSCs to produce immunosuppressive elements like indoleamine 2,3-dioxygenase (IDO), programmed death-ligand 1 (PD-L1), and prostaglandin E2 (PGE2), thereby amplifying their ability to suppress the immune response and mitigate inflammation. This process involves IFN-γ activating the JAK/STAT1/IRF1 signaling pathway within MSCs, resulting in the upregulation of PD-L1 expression. Furthermore, TNF-α can synergistically enhance the immunosuppressive effects initiated by IFN-γ in MSCs by further increasing PD-L1 expression through the NF-κB pathway. When MSCs are primed with both IFN-γ and TNF-α, they exhibit a significantly augmented immunosuppressive capability [48,49,50,51]. Table 1 summarizes the role of various events in TEC’s rejection or acceptance.

## 3. Types of Scaffolding Systems and Their Properties

The field of tissue engineering hinges upon the diverse characteristics of scaffolds, which can be broadly categorized into natural and synthetic materials, each presenting distinct advantages and limitations. While synthetic scaffolds offer facile customization and production, their biological activity tends to be limited [52]. Conversely, scaffolds produced from natural biomaterials possess superior biocompatibility [53]. On the negative side, natural biomaterials lack sufficient mechanical properties and durability [54]. Hybrid scaffolds, combining synthetic and natural polymers, represent a promising approach in tissue engineering due to their ability to synergistically harness the advantages of both material types. Synthetic polymers offer tunable mechanical properties, ease of fabrication, and precise control over scaffold architecture, while natural polymers provide inherent bioactivity and support cellular attachment and proliferation. By integrating these materials, hybrid scaffolds can mimic the complex microenvironment of native tissue more accurately, promoting cell adhesion, proliferation, and differentiation. Moreover, hybrid scaffolds can be tailored to exhibit specific degradation rates, biocompatibility, and bioactivity, making them versatile platforms for tissue regeneration applications [52,55,56]. Decellularized extracellular matrices (dECM) are highly prized in tissue engineering for their exceptional bioactivity. By preserving native ECM components like growth factors and structural proteins while removing cellular elements, dECM provides a biologically rich scaffold that supports cell adhesion, proliferation, and differentiation. This inherent bioactivity promotes cellular interactions and signaling pathways crucial for tissue regeneration. These scaffolds’ biomimetic microenvironment closely mimics native tissue, facilitating the recruitment and function of endogenous cells for tissue repair [52,57]. Recent research has debunked outdated notions surrounding the immunogenicity of ECM scaffolds, once attributed to early-generation materials crosslinked with glutaraldehyde. Contemporary dECM products, manufactured according to stringent standards, are widely regarded as safe and capable of fostering constructive tissue remodeling. FDA approval of numerous commercial products based on dECM further underscores their safety and efficacy, as corroborated by extensive research spanning over a decade [58,59,60,61].

Increasing evidence suggests that dECM can influence immune responses. Fishman et al. undertook a study where they decellularized skeletal muscle tissues and transplanted them into xenogeneic hosts to examine the cellular immune response. Their findings indicated that the decellularized scaffolds lacked major histocompatibility complex antigens and demonstrated anti-inflammatory and immunosuppressive effects. These effects were evident through delayed degradation in vivo, reduced proliferation of sensitized T cells in vitro, decreased levels of pro-inflammatory cytokines IL-2 and IFN-γ, increased levels of anti-inflammatory cytokine IL-10 in cell culture supernatants, polarization of macrophage response toward an M2 phenotype in vivo, and enhanced survival of donor-derived xenogeneic cells at 2 and 4 weeks post-transplantation. These findings suggest that decellularized scaffolds alter host responses away from a pro-inflammatory profile and may suppress T-cell responses [62].

## 4. Scaffold Designing Strategies for Modulating Inflammation

In the realm of tissue engineering, the design of scaffolds plays a pivotal role in directing the immune system’s response to the implanted structures. The inflammatory process, intricate and dynamic in nature, holds substantial sway over the efficacy of tissue regeneration. Consequently, customizing scaffold characteristics to regulate inflammatory responses becomes imperative for the development of functional TECs [24,63,64].

The selection of biomaterials for scaffolding stands out as a foundational factor influencing inflammatory reactions. Preference is often given to biocompatible and bioresorbable synthetic or natural polymers. These materials not only furnish a conducive environment for cellular activities and tissue regeneration but also mitigate adverse foreign body reactions [52]. Notably, certain biomaterials, like Hyaluronic Acid (HA) and chitosan, possess inherent anti-inflammatory properties, further enhancing their suitability for tissue engineering applications [65,66].

The characteristics of scaffold surfaces significantly impact cellular interactions and subsequent inflammatory responses. Enhancing the surface by applying immunomodulatory agents or concealing scaffold antigens with biologically inert materials can create a conducive microenvironment [52,67]. Furthermore, alterations to surface topography and roughness have the potential to influence the behavior of immune cells and, consequently, the overall inflammatory response [24].

Integrating controlled drug delivery systems into scaffolds enables the sustained release of anti-inflammatory drugs or bioactive molecules. This prolonged release can regulate the inflammatory cascade, fostering a controlled and balanced healing process. However, achieving precise control over the spatiotemporal release of these agents poses a significant challenge. To address this issue, nano- and micro-carriers have been introduced into the matrix of TECs, aiming to mitigate adverse immune reactions [67,68,69].

**Figure 2:**
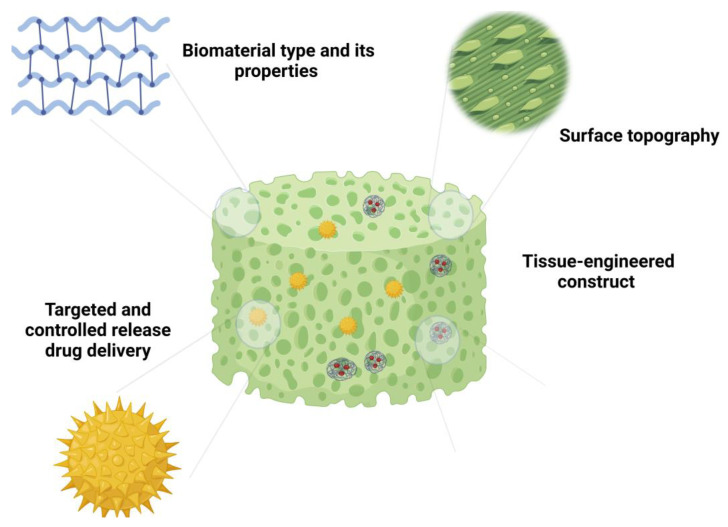
Schematic illustration representing different design strategies for modulation of inflammation.

The development of dynamic constructs that respond to the ever-changing microenvironment within host tissues represents an emerging technology. Smart materials, including stimuli-responsive hydrogels, can adapt to variations in physiological conditions [70,71]. These constructs have the potential to actively modulate inflammation by releasing therapeutic agents in response to specific cues, such as changes in pH or the presence of inflammatory mediators. In this context, Bian et al. produced pH-responsive microsphere consisting of an injectable peptide–cell–hydrogel. This was achieved through the covalent bonding of APETx2 (a peptide toxin that targets and inhibits the activity of acid-sensing ion channel 3), followed by the incorporation of nucleus pulposus cells. This microsphere demonstrates the capability to suppress local inflammatory cytokine storms, thereby regulating the metabolic equilibrium of the ECM in vitro [72]. Figure 2 shows a schematic illustration of different design strategies for inflammation modulation.

## 5. MSCs Immunomodulatory Properties: Crosstalk with the Immune System

Following the inflammatory phases of the foreign body response, regeneration becomes necessary to restore tissue function. This process relies on the self-renewal capacities of the cells located at the implant site. MSCs, also referred to as multipotential stromal cells or mesenchymal progenitor cells, are tissue-resident cells that possess self-renewal capacity along with multipotency. These cells inhabit specific tissue niches and engage in interactions with other cells to maintain a balance between proliferation, differentiation, and quiescence [73]. Therefore, MSCs influence the tissue’s ability to re-establish its complete function after tissue injury.

MSCs originating from different tissues share many similar features but differ in their expression of surface proteins as well as their proliferative and differentiation potential [74]. The International Society for Cellular Therapy (ISCT) characterizes them as expressing the canonical non-hematopoietic markers CD73, CD90, and CD105 and simultaneously lacking hematopoietic cell markers, such as CD34, CD45, CD14 or CD11b, CD19 or CD79a, HLA-DR, and CD31 [75,76]. Since MSCs display tissue-specific characteristics, the complete regeneration of the implant site depends on the tissue where the scaffold is implanted [77].

MSCs exhibit varying behaviors depending on their source, which can be bone marrow [78], adipose tissue [79], umbilical cord blood [80], placenta [81], menstrual blood [82], etc. [83]. These differences in MSCs behavior stem from the unique microenvironments and developmental origins of their respective tissues, with distinct niches in each tissue influencing their epigenetic and transcriptional profiles, thereby shaping their functional properties [48,84].

MSCs crosstalk with several immune effectors, such as T cells and macrophages, influencing tissue regeneration and scaffold outcomes [85,86]. They exert their immunomodulatory properties through direct cell-to-cell contact, the production of paracrine molecules, and the secretion of extracellular vesicles [87,88].

Following scaffold implantation, the inflammatory environment, abundant in TNF-α and IFN-γ, influences MSCs to adopt an immunomodulatory phenotype. Research indicates that MSCs rely on signals originating from the IFN-γ receptor, as decreased anti-inflammatory capabilities were observed when the IFN-γ receptor was blocked [89]. Garcia et al. incorporated IFN-γ into an injectable hydrogel to enhance the immunomodulatory properties of the encapsulated MSCs. They demonstrated that MSCs loaded into the IFN-γ tethered hydrogel exhibited a robust capacity to inhibit activated T-cell proliferation and monocyte-derived dendritic cell differentiation [90].

The initial inflammatory stimulation is useful for MSCs to fully develop their immunomodulatory potential, which involves the expression of molecules such as indoleamine 2,3-dioxygenase (IDO), programmed death ligand-1 (PD-1), IL-6, and prostaglandin E2. Additionally, MSCs produce paracrine factors such as chemokines, cytokines, and growth factors, which play a role in attracting and influencing macrophage and T-cell responses [91].

MSCs play a crucial role in aiding tissue regeneration following scaffold implantation by influencing macrophages to adopt a pro-remodeling phenotype, a critical step in resolving tissue inflammation and restoring tissue function. They achieve this by secreting immunomodulatory factors and metabolites, including insulin-like growth factor 2 (IGF-2), prostaglandin E2, TNF-stimulated gene 6 protein (TSG6P), lactate, kynurenic acid, and spermidine, which promote the polarization of macrophages into an anti-inflammatory, pro-remodeling phenotype. For example, TSG6P exhibits particularly potent immunosuppressive effects by dampening macrophage Toll-like receptor (TLR) 2/NF-kappaB responses, which in turn influence the recruitment of neutrophils and macrophages to tissue damage sites [92]. IGF-2 stimulation during macrophage maturation promotes continuous oxidative phosphorylation and consequent PD-L1 expression, features that maintain macrophage anti-inflammatory properties [93]. Extracellular vesicles, including exosomes, play a significant role in propagating the immunomodulatory effects of MSCs on macrophages. Exposing macrophages to MSC-derived exosomes has been shown to inhibit TLR4 activity and reduce macrophage inflammatory actions [94]. Another example of MSCs’ immunomodulatory role is through their production of chemokines. Chemokines such as CCL2 and CXCL12 have been found to enhance the secretion of IL-10 by macrophages. IL-10 is associated with a decrease in tissue inflammation, further highlighting the anti-inflammatory effects of MSCs [95]. Thus, MSCs play a critical role in influencing macrophage polarization and enhancing their anti-inflammatory activity. This promotes pro-remodeling responses, facilitates the restoration of tissue function, and consequently leads to improved scaffold outcomes.

MSCs also interact with cells from the adaptive immune system to maintain tissue homeostasis and restore its function. They indirectly stimulate the development of a Th2 response and facilitate the establishment of regulatory T cells by promoting a macrophage pro-remodeling phenotype. Additionally, MSCs can impede the maturation and activation of dendritic cells, thereby reducing antigen presentation to T cells and hindering T-cell activation and proliferation [96]. An array of factors produced by MSCs directly promote T-cell immunosuppressive responses. INF-γ-stimulated MSCs express IDO, which converts tryptophan into kynurenine, limiting T-cell responses. In the inflammatory microenvironment, they also upregulate heme oxygenase-1, a stress-inducible protein that boosts T regulatory cell inputs, favoring tissue regeneration rather than inflammation [97]. Prostaglandin-E2 and tumor growth factor-B are also released by MSCs and favor T regulatory responses. Furthermore, MSCs secrete chemokines, such as CXCL9, CXCL10, and CXCL11, which allows CCR3-expressing T cells to neighbor MSCs and facilitates the immunosuppressive effects of paracrinally secreted substances like nitric oxide [98], IDO-induced products [99], and secreted galectins [100]. Finally, MSCs express surface proteins that restrict T-cell inflammatory responses. IFN-γ-stimulated MSCs overexpress PD-L1, which binds to PD-1 on the surface of T cells and inhibits T-cell responses through direct cell-to-cell contact.

**Figure 3:**
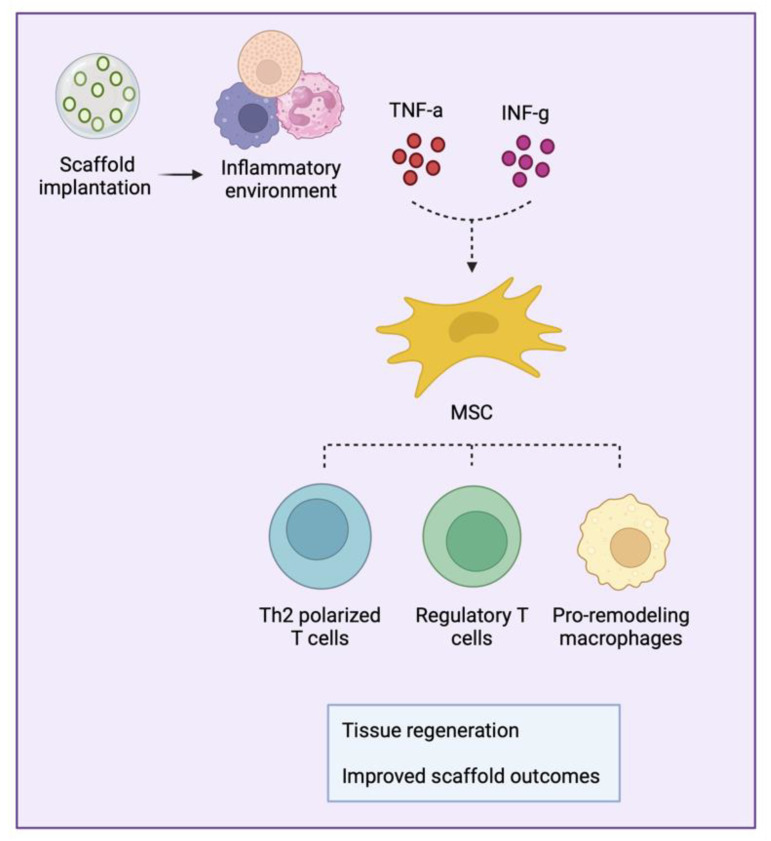
Schematic representation of mesenchymal stromal/stem cell (MSC) crosstalk with immune cells.

The immune system closely interacts with MSCs to build an appropriate environment for tissue regeneration (Figure 3). Upon activation by inflammatory cytokines, MSCs develop an immunomodulatory phenotype that facilitates and improves the healing process. In this context, the immune system responds by acquiring a regenerative profile composed of pro-remodeling macrophages, Th2-polarized T cells, and activated regulatory T cells. The orchestration of these cells promotes an adequate environment for tissue healing and the development of fully functional scaffolds.

MSCs have potent immunomodulatory properties that make them promising candidates for cell therapy, even from allogeneic sources [101]. Allogeneic MSCs possess a strong safety record and offer the advantage of being readily available for immediate use, catering to a wide range of patients. Numerous investigations have highlighted the clinical safety and prolonged efficacy of allogeneic MSCs infusions, demonstrating their ability to persist in immunocompetent hosts for more than a month. Notably, allogeneic MSCs maintain their integrity post-transplantation, showing no signs of malignant transformation [102]. Studies conducted in clinical settings have indicated that allogeneic MSCs derived from bone marrow can be safely administered to humans without causing immune reactions that are significant from a clinical perspective [103,104].

## 6. Cell–Material Interactions and How They Affect the Differentiation Program of Stem Cells

Cell–material interactions play a fundamental role in directing the differentiation program of stem cells. It has been demonstrated that several factors, including the physical and chemical properties of materials, play a major role in this process.

The differentiation of stem cells can be significantly impacted by the surface topography of biomaterials. Nanotopographical features [105], encompassing static patterned surfaces [106], dynamic patterned surfaces [106], and rough surfaces [107], have been proven to influence the adhesion, migration, proliferation, and differentiation of stem cells. Through the precise control of biomaterial topology, researchers have successfully guided the differentiation of stem cells toward specific lineages, such as osteogenic and adipogenic pathways [108]. Recent investigations highlight the fabrication of surfaces with micro/nanotopographical characteristics that emulate micro/nanoscale features, showcasing their potential to induce stem cell differentiation [109]. The interaction between cells and the surface topography is instrumental in driving stem cell fate, and extensive research has delved into the effects of nano/micrometer-scale surface topography on stem cell morphology, proliferation, migration, and differentiation [110].

Scaffolds can undergo functionalization with specific bioactive substances to replicate the native ECM. These substances encompass growth factors, cytokines, and signaling molecules that govern the differentiation of stem cells into distinct lineages [111]. An effective strategy involves creating gradients of bioactive molecules within the scaffold, directing stem cell migration and differentiation [112,113,114]. This method is particularly advantageous for constructing intricate tissues with diverse cell types and functionalities. Moreover, the integration of ECM components, such as collagen, fibronectin, and laminin, into the scaffold’s composition establishes a bioactive milieu that fosters cell adhesion and drives stem cells toward differentiation into specific cell types [115,116,117]. The hydrophilic or hydrophobic characteristics of the scaffold’s surface have also been observed to impact cell adhesion and protein adsorption, consequently influencing the behavior and differentiation of stem cells [118,119].

The mechanical properties of a material can also influence the destiny of stem cells, with cells having a tendency to specialize in lineages that align with the mechanical characteristics of their natural niche [120,121]. Substrates with different mechanical properties have the capacity to prompt the differentiation of specific cell types, exemplified in the case of MSCs differentiating into osteogenic [122], adipogenic [123], or chondrogenic lineages [124].

**Figure 4:**
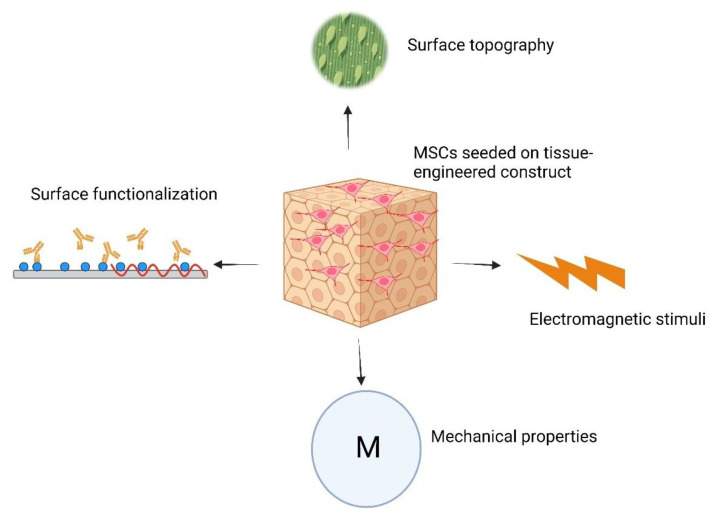
Schematic illustration representing factors influencing the differentiation program of MSCs when seeded on TECs.

Electric and electromagnetic fields have demonstrated an impact on different facets of stem cell behavior. These electrical signals have the potential to direct the differentiation of stem cells, prompting them to develop into distinct cell types, including neurons, muscle cells, and osteoblasts [125,126,127]. Figure 4 illustrates the diverse factors involved in stem cell–biomaterial interactions that influence the differentiation program of stem cells.

Culturing MSCs on TECs provides additional benefits for tissue engineering. The multilineage differentiation potential of these cells allows researchers to produce a wide range of tissues [128,129,130]. In addition, the seeded MSCs on the TECs secrete various growth factors, cytokines, and extracellular vesicles that promote tissue repair and angiogenesis [131]. Au-Yeung et al. showed that culturing MSCs on the porcine decellularized cardiac tissue resulted in increased protein density and elevated denaturation resistance [132]. Sarig et al. showed that the coculture of MSCs and human umbilical vein endothelial cells on the decellularized cardiac tissue resulted in scaffold remodeling and improved tissue integration [133]. In addition to the effects of materials on MSC’s differentiation program, it appears that MSCs demonstrate varying immunomodulation capabilities when cultured on different topographies. Wan et al. investigated this phenomenon by culturing MSCs on two distinct electrospun polylactic acid (PLLA) scaffolds. They found that the immunomodulatory behavior of these cells was notably enhanced when cultured on aligned fibers compared to random fibers. Their study revealed that FAK- and YAP/TAZ-dependent mechanotransduction pathways were responsible for the observed immunomodulation effects [134].

## 7. Summary of Previous Studies

### 7.1. Immunomodulation in Bone Tissue Engineering

Recruiting MSCs within the body for immunomodulation in TECs offers a promising alternative to conventional scaffold seeding approaches. By leveraging endogenous MSC populations, TECs can capitalize on the innate regenerative potential of these cells, avoiding exogenous cell transplantation. This approach not only simplifies TEC fabrication but also harnesses the dynamic responsiveness of resident MSCs to tissue-specific cues, enhancing their immunomodulatory functions. By modulating the local microenvironment and promoting tissue regeneration, recruited MSCs facilitate the seamless integration of TECs with host tissues, offering a sustainable and clinically relevant strategy for tissue repair [52]. In this regard, Wang et al. developed a cell-free bone scaffold by incorporating magnetic ferrite nanoparticles and lanthanum into hydroxyapatite [135]. They developed a composite material by integrating magnetic ferrite nanoparticles and lanthanum into hydroxyapatite, which was then dispersed within a chitosan matrix. Notably, the magnetic nanoparticles exhibited responsiveness to an external magnetic field, creating a conducive endogenous environment for recruiting rat bone marrow mesenchymal stem cells (BMSCs). Both in vitro and in vivo animal studies demonstrated the remarkable efficacy of the scaffold in recruiting BMSCs and promoting osteogenic differentiation. This effect was achieved through the upregulation of the phosphorylation of the Smad 1/5/9 pathway. Furthermore, the scaffold exhibited notable immunomodulatory properties, leading to the promotion of an anti-inflammatory phenotype among macrophages and immune cells.

Despite these results, recruiting endogenous MSCs for immunomodulation in TECs poses technical challenges related to the variability and heterogeneity of MSC populations within different tissue microenvironments. The efficacy of MSC recruitment strategies may be influenced by factors such as age, tissue source, and disease state, impacting the consistency and reproducibility of outcomes. Moreover, the dynamic responsiveness of resident MSCs to tissue-specific cues introduces complexity in modulating their immunomodulatory functions, necessitating precise control over signaling pathways and microenvironmental stimuli. Achieving uniform recruitment and activation of MSCs across diverse tissue contexts requires sophisticated molecular profiling and optimization of recruitment protocols, presenting significant technical hurdles in TEC development [136,137,138].

In order to investigate the impact of tissue-engineered scaffolds on osteo-immunomodulation, Wang et al. conducted a study involving the cultivation of BMSCs on laponite [139]. The results unveiled that exposure to laponite significantly enhanced the osteogenic differentiation of BMSCs, thereby triggering heightened bone formation through the activation of the oncostatin M pathway. Moreover, the cultivation of BMSCs on laponite demonstrated a remarkable ability to shift macrophage polarization from the pro-inflammatory M1 phenotype to the anti-inflammatory M2 phenotype, ultimately fostering an environment conducive to osteogenesis. Specifically, the findings indicated an increased percentage of CD206-positive macrophages, indicative of the M2 phenotype, while a substantial decrease was noted in the percentage of CCR-7-positive macrophages associated with the M1 phenotype in the presence of BMSCs cultured on laponite. Interestingly, no significant alterations in macrophage morphology were observed. The markers CCR-7 and CD11c, typically associated with M1-positive macrophages, were rarely detected, whereas a noteworthy increase in CD163 (M2)-positive cells was evident in BMSCs cultured on laponite compared to laponite alone. Furthermore, in vivo animal experiments corroborated these findings, revealing that the combination of BMSCs with laponite resulted in a diminished immune response and enhanced bone regeneration compared to laponite alone. In a separate investigation, urine-derived MSCs were cultured on a silk fibroin scaffold that underwent modification with graphene oxide and nanohydroxyapatite, aiming to enhance immunomodulation and facilitate bone regeneration [140]. The researchers postulated that the synergistic use of the scaffold and urine-derived MSCs could establish an optimal environment for both immunomodulation and the healing of bone tissue. Following the characterization of the porous scaffold and comprehensive in vitro studies, the study delved into evaluating the in vivo osteogenic properties by inducing cranial bone defects in rats. Six weeks post-implantation, a significant upswing in CD206 expression was noted, signifying an increased prevalence of M2-type macrophages. These macrophages are recognized for their role in promoting accelerated bone regeneration. However, it is noteworthy that the specific contribution of graphene oxide to these immunomodulatory properties was not explicitly addressed.

Chitin has been recognized for its acceptable cell compatibility in the realm of bone regeneration. However, the practical application of chitin-based scaffolds has faced challenges stemming from their suboptimal mechanical properties and a limited comprehension of the hydrogel–host cell interactions. In response to these limitations, a novel hybrid scaffold comprising chitin, nano-hydroxyapatite, and poly(ε-caprolactone) was developed [141]. The osteoinductive potential of this scaffold was further enhanced through the incorporation of MSCs. Comprehensive investigations into the cytocompatibility and interactions between macrophages and the hybrid scaffold, specifically in promoting angiogenesis and osteogenesis, were conducted both in in vitro and in vivo *animal* studies. The hybrid scaffold exhibited a capacity to stimulate proper osteo-differentiation through the process of endochondral ossification. Notably, gene expression analysis revealed a balanced expression of genes associated with both M1 macrophage polarization (IL-1, TNF-α, and IL-6) and M2 macrophage polarization (IL-10, Arg-1, and CCL22). One limitation of these studies could be the lack of investigation into the effect of MSC-material interaction on the immunomodulatory properties of these cells. In this context, Barzaghini et al. demonstrated that BMSCs seeded on a 3D micro-scaffold called Nichoid exhibited significantly different immunomodulatory functions compared to cells cultured on traditional culture flasks [142]. In another study, researchers cultured IFN-γ stimulated MSCs on electrospun silk fibroin scaffolds and compared the immunomodulatory activities of these cells with those cultured on glass or PLGA scaffolds. They demonstrated that MSCs cultured on silk fibroin constructs significantly reduced TNF-α secretion from lipopolysaccharide-activated murine splenocytes compared to cells cultured on other scaffolds. Thus, it appears that the immunomodulatory activity of MSCs is influenced by their surrounding microenvironment [143].

Exosomes derived from bone marrow have demonstrated osteo-immunomodulatory effects in regulating bone dynamics. Wei et al. isolated these exosomes from BMSCs undergoing osteogenic differentiation at different stages and evaluated their impact on undifferentiated BMSCs and macrophages. These exosomes reduced the expression of proinflammatory genes and markers associated with the macrophages’ M1 phenotype while enhancing the expression of early osteogenic markers in BMSCs [144].

The immunomodulatory behavior of MSCs appears to be influenced by their differentiation status. In this regard, Swartzlander et al. demonstrated that MSCs encapsulated within polyethylene glycol (PEG) hydrogel significantly dampened the foreign body response to fibroblast cell-incorporated hydrogels. They noted that the osteogenic differentiation of these cells diminished their capacity to modulate inflammation. Additionally, they identified prostaglandin E2 (PGE2) as a mediator of MSC immunomodulation of macrophages [145]. Therefore, MSC differentiation status should be taken into account when using them for immunomodulation purposes.

### 7.2. Immunomodulation in Cartilage Tissue Engineering

While BMSCs are widely acknowledged as the gold standard for cell therapy in osteoarthritis, recent reports highlight the potential benefits of synovial fluid MSCs as an alternative cell source. Li et al. delved into evaluating the immunosuppressive capabilities of synovial fluid MSCs, obtainable from the joint cavity through arthrocentesis, arthroscopy, or knee surgery [146]. Synovial fluid MSCs were cultured on 3D porous collagen and collagen/chitosan scaffolds, providing a supportive substrate for cell growth and mimicking the natural microenvironment of cartilage. The research revealed that synovial fluid MSCs cultured on 3D platforms resulted in increased expression of master gene regulators associated with the suppression of chronic inflammation (ido1, ptges, and ptgs2). Notably, even under the influence of pro-inflammatory cytokines IFN-γ and TNF-α, these cultured cells exhibited enhanced anti-inflammatory gene expression. This suggests a noteworthy potential for synovial fluid MSCs on 3D scaffolds to contribute to immunomodulation, presenting a promising avenue for their application in osteoarthritis therapy.

A groundbreaking approach was employed for the treatment of complex cartilage defects, utilizing injectable hydrogels derived from the decellularized ECM that was sourced from pig cartilage [147]. These hydrogels were specifically formulated to encapsulate urine-derived stem cells (USCs). The incorporation of USCs within the scaffold yielded notable improvements in the attachment, proliferation, and differentiation of the stem cells toward a chondrogenic lineage. Furthermore, USCs within the hydrogels exhibited heightened production of cartilage-specific ECM components, such as collagen II and aggrecan. In a rat model of cartilage defects, the USC-laden hydrogels demonstrated remarkable capabilities, including the stimulation of ECM secretion, modulation of the immune response, and promotion of cartilage regeneration. While this study highlights the positive outcomes of using USC-loaded ECM-derived hydrogels for cartilage repair, the isolation and purification process of USCs can be challenging and may result in lower cell yields or decreased cell viability compared to other sources. While whole cell-based therapy offers numerous advantages for cartilage tissue regeneration, some studies have investigated the utilization of MSC-derived nanoparticles for cartilage repair. D’Atri et al. demonstrated that MSC-derived nanoparticles effectively attenuated cartilage degeneration by virtue of their immunomodulatory properties [148].

MSCs display limited immunogenicity and release immunosuppressive soluble factors. Yang et al. delved into how scaffold configurations impacted the secretion of these substances in the context of cartilage tissue engineering. MSCs were introduced into four collagen-based scaffolds, and their growth, specialization, and secretion of immune-regulating factors were scrutinized. Three-dimensional setups, such as hydrogels and sponges, notably heightened the expression of mRNA and the production of proteins associated with immunomodulation, surpassing conventional two-dimensional setups. Moreover, supernatants from 3D setups notably inhibited the activation of allogeneic lymphocytes [149].

### 7.3. Immunomodulation in Spinal Cord Tissue Engineering

Immunomodulation plays a pivotal role in spinal cord injury repair by regulating the immune response. Balancing inflammatory reactions helps mitigate secondary damage, creating a conducive environment for neuronal survival and axonal regeneration [150]. In this context, Han et al. investigated the regenerative capabilities of BMSCs cultured on a 3D collagen scaffold, specifically focusing on neurotrophic protection and immunomodulatory mechanisms for neuronal repair [151]. In evaluating the potential benefits of using a 3D scaffold for BMSC culture in spinal cord injury (SCI) regeneration, both 3D- and 2D-cultured BMSCs were separately implanted in rats with hemisected SCI. Following transplantation, they observed a significant reduction in inflammatory cytokines (TNF-a, IL-1b, and IL-6), correlating with enhanced axonal regeneration. Despite its merits, the study has limitations, including the absence of a comprehensive exploration of long-term effects. Focusing on short-term outcomes in rats with hemisected SCI may not fully represent the complexities of human spinal cord regeneration. Additionally, the study lacks a direct comparison with other regenerative approaches or alternative cell sources, limiting the assessment of the scaffold’s superiority. Moreover, the detailed mechanisms underlying the observed reduction in inflammatory cytokines after transplantation remain elusive, warranting additional investigation for a more nuanced understanding of the potential therapeutic impact.

### 7.4. Immunomodulation in Tendon Tissue Engineering

The tendon healing process is complex, often resulting in prolonged recovery, limited regeneration, and the formation of scar tissue. To address this challenge, researchers investigated MSC-based therapy on rat Achilles tendons to modulate the inflammatory response and improve healing [152]. TNF-α-primed MSCs were seeded on a PLGA scaffold and transplanted into a rat model. After four weeks, the study found an increased concentration of type I procollagen and reduced production of the inflammatory factor IL-1α. Notably, delivering TNF-α-primed MSCs through a 3D PLGA scaffold influenced macrophage polarization and cytokine production, providing potential insights for enhancing tendon healing strategies. Russo et al. engineered a biomimetic PLGA scaffold featuring aligned fibers and investigated Amniotic Epithelial Stem Cells (AESCs) behavior on these structures. They observed that cells cultured on aligned fibers exhibited an elongated, tenocyte-like morphology. Additionally, these constructs enhanced the immunomodulatory activity of the cells, linked to the activation of the mechanotransducer YAP [153]. Alongside fiber alignment, various other scaffold characteristics, such as fiber thickness, mechanical properties, pore size, and material properties, could influence the immunomodulation of cells [52,154,155]. In this regard, Khatib et al. showed that PLGA scaffolds with different fiber sizes exhibited different effects on AESCs’ immunomodulation behavior [156]. One drawback of PLGA in tendon tissue engineering is its acidic degradation residues. Khatib et al. demonstrated that AESCs adapt their gene expression profiles in response to the acidic degradation products of PLGA, favoring an anti-inflammatory response [157].

### 7.5. Immunomodulation in Skeletal Muscle Tissue Engineering

Recent insights highlight the pivotal role of scaffold topography in influencing the paracrine functions of MSCs. In a study by Li et al., 3D polydopamine-modified bioceramics were ingeniously fabricated, featuring a uniform nanolayer pattern inspired by mussel surface coatings [158]. This nanopattern significantly augmented adipose-derived stem/stromal cells’ (ASCs) ability to modulate the immune response, fostering anti-inflammatory effects and curbing immune cell activation. The surface modification not only bolstered the secretion of various cytokines but also elevated the production of key immunomodulatory factors—COX-2, PGE-2, and TSG-6—at both mRNA and protein levels. Importantly, the study showcased a pronounced increase in the expression of CD206, an M2 marker associated with anti-inflammatory properties. Notably, when FAK or ERK1/2 suppression was introduced, there was a profound reduction in the mRNA levels of angiogenic factors such as VEGF, bFGF, HGF, and Ang-1. The collective outcome of heightened immunomodulatory and pro-angiogenic factors, coupled with the paracrine products secreted by ASC cultured on 3D bioceramics, underscores the promising therapeutic potential for skeletal muscle healing applications.

Skeletal muscle plays a vital role in the body’s physiology, yet effective treatments for volumetric muscle loss (VML) resulting from severe trauma or tumor removal remain elusive. Recent research indicates that employing a tissue engineering approach utilizing a compound comprising MSCs and a decellularized ECM scaffold yields significant regenerative effects on VML injuries. Qiu et al. demonstrated that the combination of MSCs and a decellularized ECM scaffold produces synergistic effects, notably enhancing skeletal muscle tissue regeneration [159]. Intriguingly, in the animal model, both MSCs and the decellularized ECM scaffold exhibit the ability to promote macrophage polarization toward the M2 phenotype while inhibiting polarization toward the M1 phenotype, a recognized facilitator of tissue regeneration. Of particular significance is the finding that the combined use of MSCs and the decellularized ECM scaffold produces synergistic effects on promoting macrophage polarization toward the M2 phenotype, exceeding a mere additive influence. Despite these promising results, the immunogenicity of the decellularized ECM scaffold and how it interacts with the host’s immune system need further investigation. Unanticipated immune responses or rejection could compromise the overall effectiveness of the treatment.

### 7.6. Immunomodulation in Cardiac Tissue Engineering

Immunomodulation is crucial in treating myocardial infarction as it helps regulate the immune response, minimizing excessive inflammation and promoting tissue repair. By modulating immune cells, particularly macrophages, immunomodulation assists in preventing further damage to the heart tissue post-infarction. In this regard, Monguió-Tortajada et al. evaluated the efficacy of a cellular product for myocardial infarction in a porcine model [160]. The strategy involved local delivery of EVs derived from porcine cardiac ASC. These EVs were loaded into a decellularized pericardial scaffold filled with a peptide hydrogel. Cardiac magnetic resonance imaging (MRI) showed significant enhancement in cardiac function, evidenced by improved ventricular ejection fraction and reduced ventricular dilatation after one month. Notably, a 40% reduction in scar size within the myocardium was observed, attributed to decreased levels of collagen type I and fibrotic tissue formation. This underscores the potential of EVs to mitigate adverse remodeling processes and their anti-inflammatory effects. Reduced transcription of CCL2, expression of CD163+ macrophages, and modulation of immune response factors, including IL-1, PBMC influx, TNF-α, GM-CSF levels, and CD73+ and CCR2+ monocytes, further support EV’s therapeutic potential for myocardial infarction.

Cardiac extracellular matrix (cECM) scaffolds, produced via decellularization methods, offer promise for reconstructive surgery due to their resemblance to native tissue. In this context, various methods have been developed for removing antigens from these tissues. Papalamprou et al. compared their antigen removal (AR) method with the sodium dodecyl sulfate (SDS) method for developing cECM. Comparing AR with the SDS method, they found higher MSCs infiltration in AR scaffolds, which also prompted constructive remodeling compared to SDS scaffolds, which is associated with chronic inflammation. Additionally, MSCs exhibited significantly greater immunomodulatory function on AR scaffolds compared to SDS [161]. It appears that cECM possesses an innate ability to regulate inflammatory reactions. In this context, Sarig et al. employed porcine cECM as a cardiac patch to treat myocardial infarction. These patches enhanced cardiac performance and facilitated the influx of progenitor cells expressing markers characteristic of cardiomyocytes. Furthermore, these patches underwent vascularization and bolstered regenerative remodeling, as indicated by an elevated proportion of M2/M1 macrophage phenotype at the site of injury [162]. Furthermore, cECM provides tissue-specific cues that induce the differentiation of stem cells into cardiac lineage [163,164].

### 7.7. Immunomodulation in Skin Tissue Engineering

**Figure 5:**
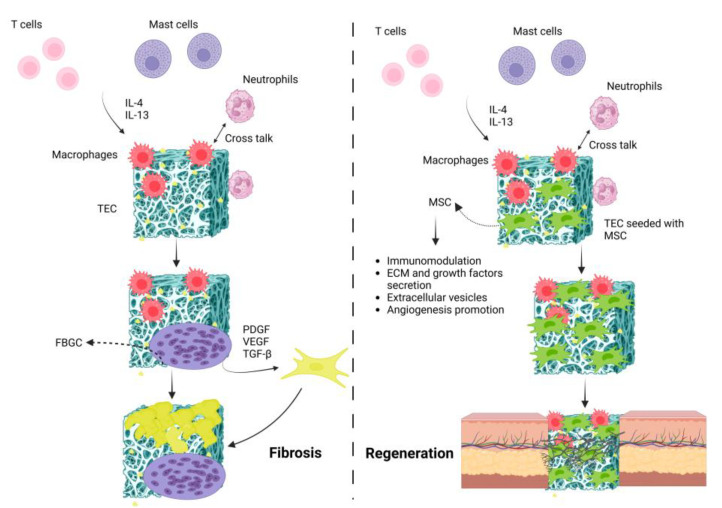
Schematic illustration representing fibrosis vs. regeneration following TEC’s implantation. In the fibrosis process, series of immunological reactions lead to the formation of foreign body giant cells (FBGCs) that result in the TEC’s fibrosis. By seeding MSCs onto the TEC, these cells modulate inflammatory responses and secrete various pro-healing factors that lead to tissue regeneration.

In situations marked by chronic wounds or impaired healing, an imbalance in the immune response can lead to prolonged inflammation or hindered tissue repair. Strategies involving immunomodulation, such as the use of cytokines, growth factors, or cell-based therapies, aim to adjust the behavior of immune cells. The goal is to establish a favorable microenvironment that supports healing, addressing the dysregulated immune response and facilitating more effective tissue repair. EV-functionalized scaffolds were developed by incorporating polyethyleneimine-modified polycaprolactone fiber [15]. These fibers, carrying a positive charge due to modification, facilitated the electrostatic tethering of negatively charged exosome membranes. Notably, the colocalization analysis revealed that exosomes predominantly interacted with CD68+ macrophages rather than CD3+ T cells. The study demonstrated that the application of exosome-functionalized scaffolds exerted significant effects on both macrophage and T-cell responses throughout the wound-healing process. Examining macrophage subtypes, the scaffolds induced the accumulation of proinflammatory M1-like macrophages (CD86+) and immunomodulatory M2-like macrophages (CD206+). A subcutaneous implantation model in mice mirrored these proinflammatory and immunomodulatory macrophage responses. Concerning T cells, the treatment facilitated CD4+ Th2 cells and CD4+CD25+FoxP3+ regulatory T cells (Tregs), leading to an increased proportion of T cells secreting the anti-inflammatory cytokine IL-10. Importantly, most CD4+ cells and all Tregs were identified as IL-10+. In a mouse model featuring large square skin excisional wounds, the utilization of exosome-functionalized scaffolds significantly expedited epidermal covering promoted the formation of thicker and healthier granulation tissues, increased collagen deposition, facilitated effective angiogenesis, and expedited wound closure. Despite being innovative, the whole cell delivery instead of EVs may provide better healing as the cells constantly modify their EV’s composition in response to their surrounding environment. Figure 5 illustrates the events leading to fibrosis or tissue regeneration followed by TEC’s implantation. Table 2 summarizes the materials used in scaffold fabrication, their modification method, and immune response to these modifications.

## 8. Challenges

In the realm of tissue regeneration, the intricate biological process of inflammation plays a dual role. While it is indispensable for initiating the repair process, the consequences can be adverse if inflammation remains uncontrolled or persists over an extended period. Such outcomes may include fibrosis, rejection, and impaired tissue regeneration. Achieving a precise balance is imperative to unlock the full potential of applications in tissue engineering.

The immunomodulatory properties of MSCs have been harnessed to modulate inflammation in TECs. However, optimizing the dosage, delivery methods, and timing of stem cell application presents challenges in maximizing the effectiveness of stem cell-mediated immunomodulation. A comprehensive understanding of these variables is crucial for refining strategies in tissue engineering. Additionally, delving into the paracrine effects of MSC, including the release of EV, poses a specific challenge. Further exploration is required to unveil the full extent of these effects and their molecular mechanisms, allowing for fine-tuning of immunomodulation in tissue engineering.

In the transplantation site, MSCs may encounter a challenging environment characterized by oxygen and nutrient deficiency. These conditions can profoundly influence their metabolic processes, potentially compromising their viability and functionality [165].

The seamless integration of MSCs into the structural framework of scaffolds emerges as a significant challenge in tissue engineering. Optimization of factors, such as cell adhesion, distribution, and viability within the TEC, is essential to enhance the overall performance of the engineered tissue.

Translating stem cell-mediated immunomodulation from preclinical studies to clinical applications is a formidable task, requiring the meticulous addressing of safety concerns, achieving scalability, and developing standardized protocols for successful and consistent clinical implementation. In addition, assessing the long-term effects of immunomodulation stands as a challenge in the field of tissue engineering. Understanding the durability of immunomodulatory effects and ensuring sustained positive outcomes over extended periods are critical.

## 9. Conclusions and Future Perspectives

In conclusion, the pivotal role of immunomodulation in the success and functionality of TECs is evident in the intricate interplay between these constructs and the host’s immune system. A well-regulated immune response is paramount for the seamless integration and sustained efficacy of engineered tissues within the complex biological milieu. This review has provided a comprehensive overview of recent advances and challenges in leveraging the immunomodulatory properties of stem/stromal cells to regulate the immune response to TECs. By synthesizing the current state of knowledge, we have highlighted the potential of these cells in modulating the host immune environment, thereby influencing the fate of implanted constructs. Looking forward, the future of tissue engineering relies heavily on refining techniques for optimal MSC delivery, dosage, and timing. Ongoing research endeavors are indispensable in unraveling the intricacies of MSC-based immunomodulation, aiming for precise control over the host immune response. This dynamic field necessitates continuous exploration to enhance our understanding and fine-tune strategies, paving the way for more effective and tailored therapeutic interventions.

The evolving insights into the interplay between MSCs and the immune system hold great promise. As our understanding deepens, it is anticipated that these advancements will lead to novel therapeutic applications, significantly expanding the scope of tissue engineering and regenerative medicine. The clinical application involving MSC-assisted tissue engineering is expanding, and we anticipate an increase in clinical trials in the near future [166]. By capitalizing on the immunomodulatory potential of MSC, we envisage groundbreaking developments that will contribute to the realization of more successful, durable, and widely applicable tissue-engineered solutions. The journey toward unlocking the full potential of immunomodulation in tissue engineering is a collaborative effort, and this review serves as a roadmap for researchers and clinicians alike, guiding them toward the next frontier of regenerative medicine.

## Figures and Tables

**Table 1:** Summary of immune response processes to TECs.

Events in Immune Responses after TEC’s Implantation	Role in TEC’s Rejection or Tissue Regeneration
Release of DAMPs	They are released by injured cells, triggering recruitment, proliferation, and activation of non-hematopoietic and hematopoietic cells, culminating in tissue repair. Outcome varies based on immune response duration and cell involvement.
Adsorption of plasma proteins to scaffold’s surface	Biomaterial properties influence protein binding on scaffold surfaces, dictating immune responses. Adsorbed proteins initiate cellular reactions, triggering inflammation and complement activation, contributing to clot formation and inflammation.
Recognition of TEC as a foreign body	Histamine, cytokines, and leukotrienes released by platelets and endothelial cells trigger neutrophil mobilization at TEC implant sites. Resident cells detect DAMPs, releasing IL-8 to attract neutrophils, initiating an inflammatory response.
Recruitment of neutrophils	Neutrophils are recruited at implant site within 72 h to combat infections by producing cytotoxic substances and reactive oxygen species. They also release neutrophil extracellular traps and IL-8, amplifying the inflammatory response and potentially degrading TEC’s surface.
Activation of mast cells	Mast cells sense biomaterial scaffolds, releasing inflammatory substances upon activation, influencing immune cell behavior, notably macrophages.
Macrophages	In the early healing stages, macrophages bind to scaffold proteins and attempt to engulf the biomaterial. The crosstalk between macrophages and neutrophils is particularly crucial for initiating tissue repair. Increased expression of phagocytic signals on the surface of dying neutrophils prompts the activation of macrophages.
Formation of FBGCs	In the chronic phase of the foreign body response, FBGCs form from fused macrophages on the implant surface. Influenced by cytokines and scaffold composition, FBGCs release substances shaping immune responses and biomaterial degradation, impacting implant outcome.
TH2 cell activation	Under IL-4 stimulation, FBGCs adopt a pro-remodeling phenotype, aiding tissue repair by boosting Th2 cell response. This activation enhances IL-4-like cytokine release, fortifying macrophage pro-remodeling traits. These stimulated macrophages, linked to improved scaffold outcomes, collaborate with fibroblasts and stem cells, fostering tissue regeneration and functional implants.

**Table 2:** Summary of scaffolds used in immunomodulation studies, their modification methods, and immune response to them.

Materials Used in Scaffold Fabrication	Modification Method	Immune Response	References
Chitosan	Integrating magnetic ferrite nanoparticles and lanthanum into hydroxyapatite.	The modification recruited MSCs at the implant site, leading to the promotion of an anti-inflammatory phenotype among macrophages and immune cells.	[135]
Laponite	Seeding with BMSCs	Cell seeding demonstrated a remarkable ability to shift macrophage polarization from the pro-inflammatory M1 phenotype to the anti-inflammatory M2 phenotype.	[139]
Silk fibroin scaffold loaded with graphene oxide and nanohydroxyapatite	Seeding with urine-derived MSCs	Six weeks post-implantation in calvarial bone, a significant upswing in CD206 expression was noted, signifying an increased prevalence of M2-type macrophages.	[140]
Chitin, nano-hydroxyapatite, and poly(ε-caprolactone)	Incorporation of MSCs	Gene expression analysis revealed a balanced expression of genes associated with both M1 macrophage polarization and M2 macrophage polarization after implantation.	[141]
Electrospun silk fibroin scaffolds	Seeding with IFN-γ stimulated MSCs	They demonstrated that MSCs cultured on silk fibroin constructs significantly reduced TNF-α secretion from lipopolysaccharide-activated murine splenocytes.	[143]
Polyethylene glycol (PEG) hydrogel	MSC encapsulation	Cell loading significantly dampened the foreign body response to fibroblast cell-incorporated hydrogels. They noted that the osteogenic differentiation of these cells diminished their capacity to modulate inflammation. Additionally, they identified prostaglandin E2 (PGE2) as a mediator of MSC immunomodulation of macrophages.	[145]
3D porous collagen and collagen/chitosan scaffolds	Incorporation of synovial fluid MSCs	Cell loading resulted in increased expression of master gene regulators associated with the suppression of chronic inflammation.	[146]
Injectable hydrogels derived from the decellularized ECM	Incorporation of urine-derived MSCs.	In a rat model of cartilage defects, the cell-laden hydrogels demonstrated remarkable capabilities, including the stimulation of ECM secretion, modulation of the immune response, and promotion of cartilage regeneration.	[147]
Collagen	Incorporation of MSCs	Three-dimensional scaffolds, such as hydrogels and sponges, notably heightened the expression of mRNA and the production of proteins associated with immunomodulation, surpassing conventional two-dimensional setups.	[149]
3D collagen scaffold	BMSC seeding	Following transplantation, they observed a significant reduction in inflammatory cytokines (TNF-a, IL-1b, and IL-6), correlating with enhanced axonal regeneration in spinal cord injury.	[151]
PLGA scaffold	Seeding with TNF-α-primed MSCs	The construct reduced production of the inflammatory factor IL-1α and influenced macrophage polarization.	[152]
PLGA scaffold	Seeding with Amniotic Epithelial Stem Cells	These constructs enhanced the immunomodulatory activity of the cells, which was linked to the activation of the mechanotransducer YAP.	[153]
Polydopamine bioceramics	Mussel surface coatings	This nanopattern significantly augmented ASC’s ability to modulate the immune response, fostering anti-inflammatory effects and curbing immune cell activation.	[158]
Decellularized ECM	MSC loading	Both MSCs and the decellularized ECM scaffold exhibited the ability to promote macrophage polarization toward the M2 phenotype while inhibiting polarization toward the M1 phenotype.	[159]
Decellularized pericardial scaffold filled with a peptide hydrogel	Loading with porcine cardiac ASC-derived extracellular vesicles	The developed scaffolds mitigated adverse remodeling processes and showed anti-inflammatory effects.	[160]
Cardiac extracellular matrix	MSCs loading	MSCs seeded onto the ECM significantly modulated inflammatory responses.	[161]
Porcine cardiac ECM	No modifications	These scaffolds underwent vascularization and bolstered regenerative remodeling, as indicated by an elevated proportion of M2/M1 macrophage phenotype at the site of injury.	[162]
Polyethyleneimine-modified polycaprolactone fibers	Surface coating with MSCs-derived exosomes	Exosomes predominantly interacted with CD68+ macrophages rather than CD3+ T cells. The scaffolds induced the accumulation of immunomodulatory M2-like macrophages.	[15]

## References

[1] Padmanabhan J., Kyriakides T.R. (2015). Nanomaterials, inflammation, and tissue engineering. Wiley Interdiscip. Rev. Nanomed. Nanobiotechnol..

[2] Crupi A., Costa A., Tarnok A., Melzer S., Teodori L. (2015). Inflammation in tissue engineering: The Janus between engraftment and rejection. Eur. J. Immunol..

[3] Sadtler K., Wolf M.T., Ganguly S., Moad C.A., Chung L., Majumdar S., Housseau F., Pardoll D.M., Elisseeff J.H. (2019). Divergent immune responses to synthetic and biological scaffolds. Biomaterials.

[4] Chung L., Maestas D.R., Housseau F., Elisseeff J.H. (2017). Key players in the immune response to biomaterial scaffolds for regenerative medicine. Adv. Drug Deliv. Rev..

[5] Saleh L.S., Bryant S.J. (2018). The host response in tissue engineering: Crosstalk between immune cells and cell-laden scaffolds. Curr. Opin. Biomed. Eng..

[6] Taraballi F., Corradetti B., Minardi S., Powel S., Cabrera F., Van Eps J.L., Weiner B.K., Tasciotti E. (2016). Biomimetic collagenous scaffold to tune inflammation by targeting macrophages. J. Tissue Eng..

[7] Song N., Scholtemeijer M., Shah K. (2020). Mesenchymal stem cell immunomodulation: Mechanisms and therapeutic potential. Trends Pharmacol. Sci..

[8] Arabpour M., Saghazadeh A., Rezaei N. (2021). Anti-inflammatory and M2 macrophage polarization-promoting effect of mesenchymal stem cell-derived exosomes. Int. Immunopharmacol..

[9] Negi N., Griffin M.D. (2020). Effects of mesenchymal stromal cells on regulatory T cells: Current understanding and clinical relevance. Stem Cells.

[10] Franquesa M., Hoogduijn M.J., Bestard O., Grinyó J.M. (2012). Immunomodulatory effect of mesenchymal stem cells on B cells. Front. Immunol..

[11] Qu X., Liu X., Cheng K., Yang R., Zhao R.C. (2012). Mesenchymal stem cells inhibit Th17 cell differentiation by IL-10 secretion. Exp. Hematol..

[12] Noh M.Y., Lim S.M., Oh K.-W., Cho K.-A., Park J., Kim K.-S., Lee S.-J., Kwon M.-S., Kim S.H. (2016). Mesenchymal stem cells modulate the functional properties of microglia via TGF-β secretion. Stem Cells Transl. Med..

[13] Zheng Y.H., Deng Y.Y., Lai W., Zheng S.Y., Bian H.N., Liu Z.A., Huang Z.F., Sun C.W., Li H.H., Luo H.M. (2018). Effect of bone marrow mesenchymal stem cells on the polarization of macrophages. Mol. Med. Rep..

[14] Zhang Q.-Z., Su W.-R., Shi S.-H., Wilder-Smith P., Xiang A.P., Wong A., Nguyen A.L., Kwon C.W., Le A.D. (2010). Human gingiva-derived mesenchymal stem cells elicit polarization of m2 macrophages and enhance cutaneous wound healing. Stem Cells.

[15] Su N., Hao Y., Wang F., Hou W., Chen H., Luo Y. (2021). Mesenchymal stromal exosome–functionalized scaffolds induce innate and adaptive immunomodulatory responses toward tissue repair. Sci. Adv..

[16] Li H., Shen S., Fu H., Wang Z., Li X., Sui X., Yuan M., Liu S., Wang G., Guo Q. (2019). Immunomodulatory functions of mesenchymal stem cells in tissue engineering. Stem Cells Int..

[17] Gonzalez-Pujana A., Vining K.H., Zhang D.K., Santos-Vizcaino E., Igartua M., Hernandez R.M., Mooney D.J. (2020). Multifunctional biomimetic hydrogel systems to boost the immunomodulatory potential of mesenchymal stromal cells. Biomaterials.

[18] Badylak S.F. (2016). TISSUE REGENERATION. A scaffold immune microenvironment. Science.

[19] Sadtler K., Estrellas K., Allen B.W., Wolf M.T., Fan H., Tam A.J., Patel C.H., Luber B.S., Wang H., Wagner K.R. (2016). Developing a pro-regenerative biomaterial scaffold microenvironment requires T helper 2 cells. Science.

[20] Du L., Wu J., Han Y., Wu C. (2024). Immunomodulatory multicellular scaffolds for tendon-to-bone regeneration. Sci. Adv..

[21] Zhao X., Li Q., Guo Z., Li Z. (2021). Constructing a cell microenvironment with biomaterial scaffolds for stem cell therapy. Stem Cell Res. Ther..

[22] Julier Z., Park A.J., Briquez P.S., Martino M.M. (2017). Promoting tissue regeneration by modulating the immune system. Acta Biomater..

[23] DeStefano S., Josyula A., Faust M., Fertil D., Lokwani R., Ngo T.B., Sadtler K. (2023). Conserved and tissue-specific immune responses to biologic scaffold implantation. bioRxiv.

[24] He J., Chen G., Liu M., Xu Z., Chen H., Yang L., Lv Y. (2020). Scaffold strategies for modulating immune microenvironment during bone regeneration. Mater. Sci. Eng. C.

[25] Karkanitsa M., Fathi P., Ngo T., Sadtler K. (2021). Mobilizing Endogenous Repair Through Understanding Immune Reaction with Biomaterials. Front. Bioeng. Biotechnol..

[26] Hu W.J., Eaton J.W., Ugarova T.P., Tang L. (2001). Molecular basis of biomaterial-mediated foreign body reactions. Blood.

[27] Anderson J.M., Rodriguez A., Chang D.T. (2008). Foreign body reaction to biomaterials. Semin. Immunol..

[28] Jenney C.R., Anderson J.M. (2000). Adsorbed serum proteins responsible for surface dependent human macrophage behavior. J. Biomed. Mater. Res..

[29] Brodbeck W.G., Colton E., Anderson J.M. (2003). Effects of adsorbed heat labile serum proteins and fibrinogen on adhesion and apoptosis of monocytes/macrophages on biomaterials. J. Mater. Sci. Mater. Med..

[30] Peiseler M., Kubes P. (2019). More friend than foe: The emerging role of neutrophils in tissue repair. J. Clin. Investig..

[31] Sousa A.B., Barbosa J.N. (2023). The Role of Neutrophils in Biomaterial-Based Tissue Repair-Shifting Paradigms. J. Funct. Biomater..

[32] Saijou E., Enomoto Y., Matsuda M., Yuet-Yin Kok C., Akira S., Tanaka M., Miyajima A. (2018). Neutrophils alleviate fibrosis in the CCl4-induced mouse chronic liver injury model. Hepatol. Commun..

[33] Vlasova I.I., Suleimanov S.K., Mikhalchik E.V., Urmantaeva N.T., Salimov E.L., Ragimov A.A., Khlebnikova T.M., Timashev P.S. (2022). Redox-Activation of Neutrophils Induced by Pericardium Scaffolds. Int. J. Mol. Sci..

[34] Selders G.S., Fetz A.E., Radic M.Z., Bowlin G.L. (2017). An overview of the role of neutrophils in innate immunity, inflammation and host-biomaterial integration. Regen. Biomater..

[35] Ozpinar E.W., Frey A.L., Cruse G., Freytes D.O. (2021). Mast cell–biomaterial interactions and tissue repair. Tissue Eng. Part B Rev..

[36] Wang R.M., Mesfin J.M., Karkanitsa M., Ungerleider J.L., Zelus E., Zhang Y., Kawakami Y., Kawakami Y., Kawakami T., Christman K.L. (2023). Immunomodulatory contribution of mast cells to the regenerative biomaterial microenvironment. NPJ Regen. Med..

[37] Wu Y., Hirschi K.K. (2021). Tissue-resident macrophage development and function. Front. Cell Dev. Biol..

[38] Hoeffel G., Ginhoux F. (2015). Ontogeny of tissue-resident macrophages. Front. Immunol..

[39] Hoeffel G., Ginhoux F. (2018). Fetal monocytes and the origins of tissue-resident macrophages. Cell. Immunol..

[40] Wynn T.A., Vannella K.M. (2016). Macrophages in Tissue Repair, Regeneration, and Fibrosis. Immunity.

[41] Soehnlein O., Lindbom L. (2010). Phagocyte partnership during the onset and resolution of inflammation. Nat. Rev.Immunol..

[42] Bouchery T., Harris N. (2019). Neutrophil-macrophage cooperation and its impact on tissue repair. Immunol. Cell Biol..

[43] Liu G., Wang J., Park Y.J., Tsuruta Y., Lorne E.F., Zhao X., Abraham E. (2008). High mobility group protein-1 inhibits phagocytosis of apoptotic neutrophils through binding to phosphatidylserine. J. Immunol..

[44] Badylak S.F., Valentin J.E., Ravindra A.K., McCabe G.P., Stewart-Akers A.M. (2008). Macrophage phenotype as a determinant of biologic scaffold remodeling. Tissue Eng. Part A.

[45] Brown B.N., Londono R., Tottey S., Zhang L., Kukla K.A., Wolf M.T., Daly K.A., Reing J.E., Badylak S.F. (2012). Macrophage phenotype as a predictor of constructive remodeling following the implantation of biologically derived surgical mesh materials. Acta Biomater..

[46] Liu Y., Suarez-Arnedo A., Shetty S., Wu Y., Schneider M., Collier J.H., Segura T. (2023). A Balance between Pro-Inflammatory and Pro-Reparative Macrophages is Observed in Regenerative D-MAPS. Adv. Sci..

[47] Li W., Liu Q., Shi J., Xu X., Xu J. (2023). The role of TNF-α in the fate regulation and functional reprogramming of mesenchymal stem cells in an inflammatory microenvironment. Front. Immunol..

[48] Pittenger M.F., Discher D.E., Péault B.M., Phinney D.G., Hare J.M., Caplan A.I. (2019). Mesenchymal stem cell perspective: Cell biology to clinical progress. NPJ Regen. Med..

[49] Yao M., Chen Z., He X., Long J., Xia X., Li Z., Yang Y., Ao L., Xing W., Lian Q. (2022). Cross talk between glucose metabolism and immunosuppression in IFN-γ–primed mesenchymal stem cells. Life Sci. Alliance.

[50] Chen Z., Yao M.-W., Shen Z.-L., Li S.-D., Xing W., Guo W., Li Z., Wu X.-F., Ao L.-Q., Lu W.-Y. (2023). Interferon-gamma and tumor necrosis factor-alpha synergistically enhance the immunosuppressive capacity of human umbilical-cord-derived mesenchymal stem cells by increasing PD-L1 expression. World J. Stem Cells.

[51] López-García L., Castro-Manrreza M.E. (2021). TNF-α and IFN-γ participate in improving the immunoregulatory capacity of mesenchymal stem/stromal cells: Importance of cell–cell contact and extracellular vesicles. Int. J. Mol. Sci..

[52] Lanza R., Langer R., Vacanti J.P., Atala A. (2020). Principles of Tissue Engineering.

[53] Ullah S., Chen X. (2020). Fabrication, applications and challenges of natural biomaterials in tissue engineering. Appl. Mater. Today.

[54] Abdelbasset W.K., Alrawaili S.M., Osailan A.M., Abdelmoniem Ibrahim A., Soliman G.S., Abodonya A.M. (2022). Polysaccharides, as biological macromolecule-based scaffolding systems in heart valve tissue engineering: A review. Cellulose.

[55] Wasyłeczko M., Sikorska W., Chwojnowski A. (2020). Review of synthetic and hybrid scaffolds in cartilage tissue engineering. Membranes.

[56] Wolfe P.S., Sell S.A., Bowlin G.L. (2010). Natural and synthetic scaffolds. Tissue Engineering: From Lab to Clinic.

[57] Zhang X., Chen X., Hong H., Hu R., Liu J., Liu C. (2022). Decellularized extracellular matrix scaffolds: Recent trends and emerging strategies in tissue engineering. Bioact. Mater..

[58] Golebiowska A.A., Intravaia J.T., Sathe V.M., Kumbar S.G., Nukavarapu S.P. (2024). Decellularized extracellular matrix biomaterials for regenerative therapies: Advances, challenges and clinical prospects. Bioact. Mater..

[59] Heath D.E. (2019). A review of decellularized extracellular matrix biomaterials for regenerative engineering applications. Regen. Eng. Transl. Med..

[60] Hernandez M.J., Yakutis G.E., Zelus E.I., Hill R.C., Dzieciatkowska M., Hansen K.C., Christman K.L. (2020). Manufacturing considerations for producing and assessing decellularized extracellular matrix hydrogels. Methods.

[61] Parmaksiz M., Dogan A., Odabas S., Elçin A.E., Elçin Y.M. (2016). Clinical applications of decellularized extracellular matrices for tissue engineering and regenerative medicine. Biomed. Mater..

[62] Fishman J.M., Lowdell M.W., Urbani L., Ansari T., Burns A.J., Turmaine M., North J., Sibbons P., Seifalian A.M., Wood K.J. (2013). Immunomodulatory effect of a decellularized skeletal muscle scaffold in a discordant xenotransplantation model. Proc. Natl. Acad. Sci. USA.

[63] Boehler R.M., Graham J.G., Shea L.D. (2011). Tissue engineering tools for modulation of the immune response. Biotechniques.

[64] Hotaling N.A., Tang L., Irvine D.J., Babensee J.E. (2015). Biomaterial strategies for immunomodulation. Annu. Rev. Biomed. Eng..

[65] Altman R., Bedi A., Manjoo A., Niazi F., Shaw P., Mease P. (2019). Anti-inflammatory effects of intra-articular hyaluronic acid: A systematic review. Cartilage.

[66] Chang S.-H., Lin Y.-Y., Wu G.-J., Huang C.-H., Tsai G.J. (2019). Effect of chitosan molecular weight on anti-inflammatory activity in the RAW 264.7 macrophage model. Int. J. Biol. Macromol..

[67] Antmen E., Vrana N.E., Hasirci V. (2021). The role of biomaterials and scaffolds in immune responses in regenerative medicine: Macrophage phenotype modulation by biomaterial properties and scaffold architectures. Biomater. Sci..

[68] Fan Z., Li P.Y., Deng J., Bady S.C., Cheng H. (2018). Cell membrane coating for reducing nanoparticle-induced inflammatory responses to scaffold constructs. Nano Res..

[69] Li R., Liang J., He Y., Qin J., He H., Lee S., Pang Z., Wang J. (2018). Sustained release of immunosuppressant by nanoparticle-anchoring hydrogel scaffold improved the survival of transplanted stem cells and tissue regeneration. Theranostics.

[70] Sood N., Bhardwaj A., Mehta S., Mehta A. (2016). Stimuli-responsive hydrogels in drug delivery and tissue engineering. Drug Deliv..

[71] Askari E., Seyfoori A., Amereh M., Gharaie S.S., Ghazali H.S., Ghazali Z.S., Khunjush B., Akbari M. (2020). Stimuli-responsive hydrogels for local post-surgical drug delivery. Gels.

[72] Bian J., Cai F., Chen H., Tang Z., Xi K., Tang J., Wu L., Xu Y., Deng L., Gu Y. (2021). Modulation of local overactive inflammation via injectable hydrogel microspheres. Nano Lett..

[73] Moore K.A., Lemischka I.R. (2006). Stem cells and their niches. Science.

[74] Li P., Ou Q., Shi S., Shao C. (2023). Immunomodulatory properties of mesenchymal stem cells/dental stem cells and their therapeutic applications. Cell Mol. Immunol..

[75] Stamnitz S., Klimczak A. (2021). Mesenchymal Stem Cells, Bioactive Factors, and Scaffolds in Bone Repair: From Research Perspectives to Clinical Practice. Cells.

[76] Dominici M., Le Blanc K., Mueller I., Slaper-Cortenbach I., Marini F., Krause D., Deans R., Keating A., Prockop D., Horwitz E. (2006). Minimal criteria for defining multipotent mesenchymal stromal cells. The International Society for Cellular Therapy position statement. Cytotherapy.

[77] Kozlowska U., Krawczenko A., Futoma K., Jurek T., Rorat M., Patrzalek D., Klimczak A. (2019). Similarities and differences between mesenchymal stem/progenitor cells derived from various human tissues. World J. Stem Cells.

[78] Takam Kamga P., Bazzoni R., Dal Collo G., Cassaro A., Tanasi I., Russignan A., Tecchio C., Krampera M. (2021). The role of notch and wnt signaling in MSC communication in normal and leukemic bone marrow niche. Front. Cell Dev. Biol..

[79] Alexandrushkina N., Nimiritsky P., Eremichev R., Popov V., Arbatskiy M., Danilova N., Malkov P., Akopyan Z., Tkachuk V., Makarevich P. (2020). Cell sheets from adipose tissue MSC induce healing of pressure ulcer and prevent fibrosis via trigger effects on granulation tissue growth and vascularization. Int. J. Mol. Sci..

[80] Um S., Ha J., Choi S.J., Oh W., Jin H.J. (2020). Prospects for the therapeutic development of umbilical cord blood-derived mesenchymal stem cells. World J. Stem Cells.

[81] Papait A., Vertua E., Magatti M., Ceccariglia S., De Munari S., Silini A.R., Sheleg M., Ofir R., Parolini O. (2020). Mesenchymal stromal cells from fetal and maternal placenta possess key similarities and differences: Potential implications for their applications in regenerative medicine. Cells.

[82] Farzamfar S., Salehi M., Ehterami A., Naseri-Nosar M., Vaez A., Zarnani A.H., Sahrapeyma H., Shokri M.-R., Aleahmad M. (2018). Promotion of excisional wound repair by a menstrual blood-derived stem cell-seeded decellularized human amniotic membrane. Biomed. Eng. Lett..

[83] Jin Y., Li S., Yu Q., Chen T., Liu D. (2023). Application of stem cells in regeneration medicine. MedComm.

[84] Fonseca L.N., Bolívar-Moná S., Agudelo T., Beltrán L.D., Camargo D., Correa N., Del Castillo M.A., de Castro S.F., Fula V., García G. (2023). Cell surface markers for mesenchymal stem cells related to the skeletal system: A scoping review. Heliyon.

[85] Klimczak A., Kozlowska U. (2016). Mesenchymal Stromal Cells and Tissue-Specific Progenitor Cells: Their Role in Tissue Homeostasis. Stem Cells Int..

[86] Krampera M., Le Blanc K. (2021). Mesenchymal stromal cells: Putative microenvironmental modulators become cell therapy. Cell Stem Cell.

[87] Wang Y., Fang J., Liu B., Shao C., Shi Y. (2022). Reciprocal regulation of mesenchymal stem cells and immune responses. Cell Stem Cell.

[88] English K., Ryan J.M., Tobin L., Murphy M.J., Barry F.P., Mahon B.P. (2009). Cell contact, prostaglandin E_2_ and transforming growth factor beta 1 play non-redundant roles in human mesenchymal stem cell induction of CD4^+^CD25^High^ forkhead box P3^+^ regulatory T cells. Clin. Exp. Immunol..

[89] Sheng H., Wang Y., Jin Y., Zhang Q., Zhang Y., Wang L., Shen B., Yin S., Liu W., Cui L. (2008). A critical role of IFNgamma in priming MSC-mediated suppression of T cell proliferation through up-regulation of B7-H1. Cell Res..

[90] García J.R., Quirós M., Han W.M., O’Leary M.N., Cox G.N., Nusrat A., García A.J. (2019). IFN-γ-tethered hydrogels enhance mesenchymal stem cell-based immunomodulation and promote tissue repair. Biomaterials.

[91] Shi Y., Wang Y., Li Q., Liu K., Hou J., Shao C., Wang Y. (2018). Immunoregulatory mechanisms of mesenchymal stem and stromal cells in inflammatory diseases. Nat. Rev. Nephrol..

[92] Wang G., Cao K., Liu K., Xue Y., Roberts A.I., Li F., Han Y., Rabson A.B., Wang Y., Shi Y. (2018). Kynurenic acid, an IDO metabolite, controls TSG-6-mediated immunosuppression of human mesenchymal stem cells. Cell Death Differ..

[93] Du L., Lin L., Li Q., Liu K., Huang Y., Wang X., Cao K., Chen X., Cao W., Li F. (2019). IGF-2 Preprograms Maturing Macrophages to Acquire Oxidative Phosphorylation-Dependent Anti-inflammatory Properties. Cell Metab..

[94] Zhao J., Li X., Hu J., Chen F., Qiao S., Sun X., Gao L., Xie J., Xu B. (2019). Mesenchymal stromal cell-derived exosomes attenuate myocardial ischaemia-reperfusion injury through miR-182-regulated macrophage polarization. Cardiovasc. Res..

[95] Giri J., Das R., Nylen E., Chinnadurai R., Galipeau J. (2020). CCL2 and CXCL12 Derived from Mesenchymal Stromal Cells Cooperatively Polarize IL-10+ Tissue Macrophages to Mitigate Gut Injury. Cell Rep..

[96] Chiesa S., Morbelli S., Morando S., Massollo M., Marini C., Bertoni A., Frassoni F., Bartolome S.T., Sambuceti G., Traggiai E. (2011). Mesenchymal stem cells impair in vivo T-cell priming by dendritic cells. Proc. Natl. Acad. Sci. USA.

[97] Mougiakakos D., Jitschin R., Johansson C.C., Okita R., Kiessling R., Le Blanc K. (2011). The impact of inflammatory licensing on heme oxygenase-1-mediated induction of regulatory T cells by human mesenchymal stem cells. Blood.

[98] Ren G., Zhang L., Zhao X., Xu G., Zhang Y., Roberts A.I., Zhao R.C., Shi Y. (2008). Mesenchymal stem cell-mediated immunosuppression occurs via concerted action of chemokines and nitric oxide. Cell Stem Cell.

[99] Su J., Chen X., Huang Y., Li W., Li J., Cao K., Cao G., Zhang L., Li F., Roberts A.I. (2014). Phylogenetic distinction of iNOS and IDO function in mesenchymal stem cell-mediated immunosuppression in mammalian species. Cell Death Differ..

[100] Sioud M., Mobergslien A., Boudabous A., Floisand Y. (2011). Mesenchymal stem cell-mediated T cell suppression occurs through secreted galectins. Int. J. Oncol..

[101] Nasef A., Ashammakhi N., Fouillard L. (2008). Immunomodulatory effect of mesenchymal stromal cells: Possible mechanisms. Regen. Med..

[102] Oliva A.A., McClain-Moss L., Pena A., Drouillard A., Hare J.M. (2019). Allogeneic mesenchymal stem cell therapy: A regenerative medicine approach to geroscience. Aging Med..

[103] Hare J.M., Fishman J.E., Gerstenblith G., Velazquez D.L.D., Zambrano J.P., Suncion V.Y., Tracy M., Ghersin E., Johnston P.V., Brinker J.A. (2012). Comparison of allogeneic vs autologous bone marrow–derived mesenchymal stem cells delivered by transendocardial injection in patients with ischemic cardiomyopathy: The POSEIDON randomized trial. JAMA.

[104] Karantalis V., Schulman I.H., Balkan W., Hare J.M. (2015). Allogeneic cell therapy: A new paradigm in therapeutics. Am. Heart Assoc..

[105] McNamara L.E., McMurray R.J., Biggs M.J., Kantawong F., Oreffo R.O., Dalby M.J. (2010). Nanotopographical control of stem cell differentiation. J. Tissue Eng..

[106] Gong T., Zhao K., Yang G., Li J., Chen H., Chen Y., Zhou S. (2014). The control of mesenchymal stem cell differentiation using dynamically tunable surface microgrooves. Adv. Healthc. Mater..

[107] Boyan B., Cheng A., Olivares-Navarrete R., Schwartz Z. (2016). Implant surface design regulates mesenchymal stem cell differentiation and maturation. Adv. Dent. Res..

[108] Abagnale G., Steger M., Nguyen V.H., Hersch N., Sechi A., Joussen S., Denecke B., Merkel R., Hoffmann B., Dreser A. (2015). Surface topography enhances differentiation of mesenchymal stem cells towards osteogenic and adipogenic lineages. Biomaterials.

[109] Prasopthum A., Cooper M., Shakesheff K.M., Yang J. (2019). Three-dimensional printed scaffolds with controlled micro-/nanoporous surface topography direct chondrogenic and osteogenic differentiation of mesenchymal stem cells. ACS Appl. Mater. Interfaces.

[110] Xiao L., Sun Y., Liao L., Su X. (2023). Response of mesenchymal stem cells to surface topography of scaffolds and the underlying mechanisms. J. Mater. Chem. B.

[111] Xing F., Li L., Zhou C., Long C., Wu L., Lei H., Kong Q., Fan Y., Xiang Z., Zhang X. (2019). Regulation and directing stem cell fate by tissue engineering functional microenvironments: Scaffold physical and chemical cues. Stem Cells Int..

[112] Tong X., Jiang J., Zhu D., Yang F. (2016). Hydrogels with dual gradients of mechanical and biochemical cues for deciphering cell-niche interactions. ACS Biomater. Sci. Eng..

[113] Wan X., Liu Z., Li L. (2021). Manipulation of stem cells fates: The master and multifaceted roles of biophysical cues of biomaterials. Adv. Funct. Mater..

[114] Tan K.Y., Lin H., Ramstedt M., Watt F.M., Huck W.T., Gautrot J.E. (2013). Decoupling geometrical and chemical cues directing epidermal stem cell fate on polymer brush-based cell micro-patterns. Integr. Biol..

[115] Chen Y., Lee K., Kawazoe N., Yang Y., Chen G. (2020). ECM scaffolds mimicking extracellular matrices of endochondral ossification for the regulation of mesenchymal stem cell differentiation. Acta Biomater..

[116] Chastain S.R., Kundu A.K., Dhar S., Calvert J.W., Putnam A.J. (2006). Adhesion of mesenchymal stem cells to polymer scaffolds occurs via distinct ECM ligands and controls their osteogenic differentiation. J. Biomed. Mater. Res. Part A Off. J. Soc. Biomater. Jpn. Soc. Biomater. Aust. Soc. Biomater. Korean Soc. Biomater..

[117] Mao Y., Hoffman T., Wu A., Goyal R., Kohn J. (2017). Cell type–specific extracellular matrix guided the differentiation of human mesenchymal stem cells in 3D polymeric scaffolds. J. Mater. Sci. Mater. Med..

[118] Xing Z., Cai J., Sun Y., Cao M., Li Y., Xue Y., Finne-Wistrand A., Kamal M. (2020). Altered surface hydrophilicity on copolymer scaffolds stimulate the osteogenic differentiation of human mesenchymal stem cells. Polymers.

[119] Ghasemi-Mobarakeh L., Prabhakaran M.P., Tian L., Shamirzaei-Jeshvaghani E., Dehghani L., Ramakrishna S. (2015). Structural properties of scaffolds: Crucial parameters towards stem cells differentiation. World J. Stem Cells.

[120] Wang S., Hashemi S., Stratton S., Arinzeh T.L. (2021). The effect of physical cues of biomaterial scaffolds on stem cell behavior. Adv. Healthc. Mater..

[121] Li J., Liu Y., Zhang Y., Yao B., Enhejirigala, Li Z., Song W., Wang Y., Duan X., Yuan X. (2021). Biophysical and biochemical cues of biomaterials guide mesenchymal stem cell behaviors. Front. Cell Dev. Biol..

[122] Liu Y., Yang G., Ji H., Xiang T., Luo E., Zhou S. (2017). Synergetic effect of topological cue and periodic mechanical tension-stress on osteogenic differentiation of rat bone mesenchymal stem cells. Colloids Surf. B Biointerfaces.

[123] Young D.A., Choi Y.S., Engler A.J., Christman K.L. (2013). Stimulation of adipogenesis of adult adipose-derived stem cells using substrates that mimic the stiffness of adipose tissue. Biomaterials.

[124] Panadero J., Lanceros-Mendez S., Ribelles J.G. (2016). Differentiation of mesenchymal stem cells for cartilage tissue engineering: Individual and synergetic effects of three-dimensional environment and mechanical loading. Acta Biomater..

[125] Wang Y., Wang Q., Luo S., Chen Z., Zheng X., Kankala R.K., Chen A., Wang S. (2021). 3D bioprinting of conductive hydrogel for enhanced myogenic differentiation. Regen. Biomater..

[126] Rahmani A., Nadri S., Kazemi H.S., Mortazavi Y., Sojoodi M. (2019). Conductive electrospun scaffolds with electrical stimulation for neural differentiation of conjunctiva mesenchymal stem cells. Artif. Organs.

[127] Li J., Liu X., Crook J.M., Wallace G.G. (2020). Electrical stimulation-induced osteogenesis of human adipose derived stem cells using a conductive graphene-cellulose scaffold. Mater. Sci. Eng. C.

[128] Rosenbaum A.J., Grande D.A., Dines J.S. (2008). The use of mesenchymal stem cells in tissue engineering: A global assessment. Organogenesis.

[129] Wang Y., Bronshtein T., Sarig U., Nguyen E.B.-V., Boey F.Y.C., Venkatraman S.S., Machluf M. (2013). A mathematical model predicting the coculture dynamics of endothelial and mesenchymal stem cells for tissue regeneration. Tissue Eng. Part A.

[130] Sarig U., Nguyen E.B.-V., Wang Y., Ting S., Bronshtein T., Sarig H., Dahan N., Gvirtz M., Reuveny S., Oh S.K. (2015). Pushing the envelope in tissue engineering: Ex vivo production of thick vascularized cardiac extracellular matrix constructs. Tissue Eng. Part A.

[131] Sevivas N., Teixeira F.G., Portugal R., Direito-Santos B., Espregueira-Mendes J., Oliveira F.J., Silva R.F., Sousa N., Sow W.T., Nguyen L.T. (2018). Mesenchymal stem cell secretome improves tendon cell viability in vitro and tendon-bone healing in vivo when a tissue engineering strategy is used in a rat model of chronic massive rotator cuff tear. Am. J. Sports Med..

[132] Au-Yeung G.C.T., Sarig U., Sarig H., Bogireddi H., Bronshtein T., Baruch L., Spizzichino A., Bortman J., Freddy B.Y.C., Machluf M. (2017). Restoring the biophysical properties of decellularized patches through recellularization. Biomater. Sci..

[133] Sarig U., Sarig H., Gora A., Krishnamoorthi M.K., Au-Yeung G.C.T., de-Berardinis E., Chaw S.Y., Mhaisalkar P., Bogireddi H., Ramakrishna S. (2018). Biological and mechanical interplay at the Macro-and Microscales Modulates the Cell-Niche Fate. Sci. Rep..

[134] Wan S., Fu X., Ji Y., Li M., Shi X., Wang Y. (2018). FAK-and YAP/TAZ dependent mechanotransduction pathways are required for enhanced immunomodulatory properties of adipose-derived mesenchymal stem cells induced by aligned fibrous scaffolds. Biomaterials.

[135] Wang Q., Tang Y., Ke Q., Yin W., Zhang C., Guo Y., Guan J. (2020). Magnetic lanthanum-doped hydroxyapatite/chitosan scaffolds with endogenous stem cell-recruiting and immunomodulatory properties for bone regeneration. J. Mater. Chem. B.

[136] Andreas K., Sittinger M., Ringe J. (2014). Toward in situ tissue engineering: Chemokine-guided stem cell recruitment. Trends Biotechnol..

[137] Ko I.K., Lee S.J., Atala A., Yoo J.J. (2013). In situ tissue regeneration through host stem cell recruitment. Exp. Mol. Med..

[138] Vanden Berg-Foels W.S. (2014). In situ tissue regeneration: Chemoattractants for endogenous stem cell recruitment. Tissue Eng. Part B Rev..

[139] Li T., Liu Z.L., Xiao M., Yang Z.Z., Peng M.Z., Li C.D., Zhou X.J., Wang J.W. (2018). Impact of bone marrow mesenchymal stem cell immunomodulation on the osteogenic effects of laponite. Stem Cell Res. Ther..

[140] Sun J., Li L., Xing F., Yang Y., Gong M., Liu G., Wu S., Luo R., Duan X., Liu M. (2021). Graphene oxide-modified silk fibroin/nanohydroxyapatite scaffold loaded with urine-derived stem cells for immunomodulation and bone regeneration. Stem Cell Res. Ther..

[141] Ji X., Yuan X., Ma L., Bi B., Zhu H., Lei Z., Liu W., Pu H., Jiang J., Jiang X. (2020). Mesenchymal stem cell-loaded thermosensitive hydroxypropyl chitin hydrogel combined with a three-dimensional-printed poly (ε-caprolactone)/nano-hydroxyapatite scaffold to repair bone defects via osteogenesis, angiogenesis and immunomodulation. Theranostics.

[142] Barzaghini B., Carelli S., Messa L., Rey F., Avanzini M.A., Jacchetti E., Maghraby E., Berardo C., Zuccotti G., Raimondi M.T. (2023). Bone marrow mesenchymal stem cells expanded inside the Nichoid micro-scaffold: A focus on anti-inflammatory response. Regen. Eng. Transl. Med..

[143] Kim O.-H., Yoon O.J., Lee H.J. (2019). Silk fibroin scaffolds potentiate immunomodulatory function of human mesenchymal stromal cells. Biochem. Biophys. Res. Commun..

[144] Wei F., Li Z., Crawford R., Xiao Y., Zhou Y. (2019). Immunoregulatory role of exosomes derived from differentiating mesenchymal stromal cells on inflammation and osteogenesis. J. Tissue Eng. Regen. Med..

[145] Swartzlander M.D., Blakney A.K., Amer L.D., Hankenson K.D., Kyriakides T.R., Bryant S.J. (2015). Immunomodulation by mesenchymal stem cells combats the foreign body response to cell-laden synthetic hydrogels. Biomaterials.

[146] Li J., Chen T., Huang X., Zhao Y., Wang B., Yin Y., Cui Y., Zhao Y., Zhang R., Wang X. (2018). Substrate-independent immunomodulatory characteristics of mesenchymal stem cells in three-dimensional culture. PLoS ONE.

[147] Zeng J., Huang L., Xiong H., Li Q., Wu C., Huang Y., Xie H., Shen B. (2022). Injectable decellularized cartilage matrix hydrogel encapsulating urine-derived stem cells for immunomodulatory and cartilage defect regeneration. NPJ Regen. Med..

[148] D’Atri D., Zerrillo L., Garcia J., Oieni J., Lupu-Haber Y., Schomann T., Chan A., Cruz L., Creemers L., Machluf M. (2021). Nanoghosts: Mesenchymal Stem cells derived nanoparticles as a unique approach for cartilage regeneration. J. Control. Release.

[149] Yang J., Chen X., Yuan T., Yang X., Fan Y., Zhang X. (2017). Regulation of the secretion of immunoregulatory factors of mesenchymal stem cells (MSCs) by collagen-based scaffolds during chondrogenesis. Mater. Sci. Eng. C.

[150] Pang Q.-M., Chen S.-Y., Fu S.-P., Zhou H., Zhang Q., Ao J., Luo X.-P., Zhang T. (2022). Regulatory role of mesenchymal stem cells on secondary inflammation in spinal cord injury. J. Inflamm. Res..

[151] Han S., Wang B., Li X., Xiao Z., Han J., Zhao Y., Fang Y., Yin Y., Chen B., Dai J. (2016). Bone marrow-derived mesenchymal stem cells in three-dimensional culture promote neuronal regeneration by neurotrophic protection and immunomodulation. J. Biomed. Mater. Res. Part A.

[152] Aktas E., Chamberlain C.S., Saether E.E., Duenwald-Kuehl S.E., Kondratko-Mittnacht J., Stitgen M., Lee J.S., Clements A.E., Murphy W.L., Vanderby R. (2017). Immune modulation with primed mesenchymal stem cells delivered via biodegradable scaffold to repair an Achilles tendon segmental defect. J. Orthop. Res..

[153] Russo V., El Khatib M., Prencipe G., Mauro A., Di Giacinto O., Haidar-Montes A.A., Pulcini F., Dufrusine B., Cerveró-Varona A., Faydaver M. (2022). Tendon 3D scaffolds establish a tailored microenvironment instructing paracrine mediated regenerative amniotic epithelial stem cells potential. Biomedicines.

[154] Rahmati M., Mills D.K., Urbanska A.M., Saeb M.R., Venugopal J.R., Ramakrishna S., Mozafari M. (2021). Electrospinning for tissue engineering applications. Prog. Mater. Sci..

[155] Owida H.A., Al-Nabulsi J.I., Alnaimat F., Al-Ayyad M., Turab N.M., Al Sharah A., Shakur M. (2022). Recent applications of electrospun nanofibrous scaffold in tissue engineering. Appl. Bionics Biomech..

[156] El Khatib M., Mauro A., Di Mattia M., Wyrwa R., Schweder M., Ancora M., Lazzaro F., Berardinelli P., Valbonetti L., Di Giacinto O. (2020). Electrospun PLGA fiber diameter and alignment of tendon biomimetic fleece potentiate tenogenic differentiation and immunomodulatory function of amniotic epithelial stem cells. Cells.

[157] El Khatib M., Russo V., Prencipe G., Mauro A., Wyrwa R., Grimm G., Di Mattia M., Berardinelli P., Schnabelrauch M., Barboni B. (2021). Amniotic epithelial stem cells counteract acidic degradation by-products of electrospun PLGA scaffold by improving their immunomodulatory profile in vitro. Cells.

[158] Li T., Ma H., Ma H., Ma Z., Qiang L., Yang Z., Yang X., Zhou X., Dai K., Wang J. (2019). Mussel-inspired nanostructures potentiate the immunomodulatory properties and angiogenesis of mesenchymal stem cells. ACS Appl. Mater. Interfaces.

[159] Qiu X., Liu S., Zhang H., Zhu B., Su Y., Zheng C., Tian R., Wang M., Kuang H., Zhao X. (2018). Mesenchymal stem cells and extracellular matrix scaffold promote muscle regeneration by synergistically regulating macrophage polarization toward the M2 phenotype. Stem Cell Res. Ther..

[160] Monguió-Tortajada M., Prat-Vidal C., Martínez-Falguera D., Teis A., Soler-Botija C., Courageux Y., Munizaga-Larroudé M., Moron-Font M., Bayes-Genis A., Borràs F.E. (2022). Acellular cardiac scaffolds enriched with MSC-derived extracellular vesicles limit ventricular remodelling and exert local and systemic immunomodulation in a myocardial infarction porcine model. Theranostics.

[161] Papalamprou A., Chang C.W., Vapniarsky N., Clark A., Walker N., Griffiths L.G. (2016). Xenogeneic cardiac extracellular matrix scaffolds with or without seeded mesenchymal stem cells exhibit distinct in vivo immunosuppressive and regenerative properties. Acta Biomater..

[162] Sarig U., Sarig H., de-Berardinis E., Chaw S.-Y., Nguyen E.B., Ramanujam V.S., Thang V.D., Al-Haddawi M., Liao S., Seliktar D. (2016). Natural myocardial ECM patch drives cardiac progenitor based restoration even after scarring. Acta Biomater..

[163] Krishnamoorthi M.K., Sarig U., Baruch L., Ting S., Reuveny S., Oh S., Goldfracht I., Gepstein L., Venkatraman S.S., Tan L.P. (2020). Robust fabrication of composite 3D scaffolds with tissue-specific bioactivity: A proof-of-concept Study. ACS Appl. Bio Mater..

[164] Davidov T., Efraim Y., Hayam R., Oieni J., Baruch L., Machluf M. (2021). Extracellular matrix hydrogels originated from different organs mediate tissue-specific properties and function. Int. J. Mol. Sci..

[165] Salazar-Noratto G.E., Luo G., Denoeud C., Padrona M., Moya A., Bensidhoum M., Bizios R., Potier E., Logeart-Avramoglou D., Petite H. (2020). Understanding and leveraging cell metabolism to enhance mesenchymal stem cell transplantation survival in tissue engineering and regenerative medicine applications. Stem Cells.

[166] Venkataiah V.S., Yahata Y., Kitagawa A., Inagaki M., Kakiuchi Y., Nakano M., Suzuki S., Handa K., Saito M. (2021). Clinical applications of cell-scaffold constructs for bone regeneration therapy. Cells.

